# Clinical and biochemical associations of urinary metabolites: quantitative epidemiological approach on renal-cardiometabolic biomarkers

**DOI:** 10.1093/ije/dyad162

**Published:** 2023-11-29

**Authors:** Tianqi Li, Andrei Ihanus, Pauli Ohukainen, Marjo-Riitta Järvelin, Mika Kähönen, Johannes Kettunen, Olli T Raitakari, Terho Lehtimäki, Ville-Petteri Mäkinen, Tuulia Tynkkynen, Mika Ala-Korpela

**Affiliations:** Systems Epidemiology, Faculty of Medicine, University of Oulu, Oulu, Finland; Research Unit of Population Health, Faculty of Medicine, University of Oulu, Oulu, Finland; Biocenter Oulu, University of Oulu, Oulu, Finland; Systems Epidemiology, Faculty of Medicine, University of Oulu, Oulu, Finland; Research Unit of Population Health, Faculty of Medicine, University of Oulu, Oulu, Finland; Biocenter Oulu, University of Oulu, Oulu, Finland; NMR Metabolomics Laboratory, School of Pharmacy, University of Eastern Finland, Kuopio, Finland; Systems Epidemiology, Faculty of Medicine, University of Oulu, Oulu, Finland; Research Unit of Population Health, Faculty of Medicine, University of Oulu, Oulu, Finland; Biocenter Oulu, University of Oulu, Oulu, Finland; Research Unit of Population Health, Faculty of Medicine, University of Oulu, Oulu, Finland; Unit of Primary Health Care, Oulu University Hospital, OYS, Oulu, Finland; Department of Epidemiology and Biostatistics, MRC-PHE Centre for Environment and Health, Imperial College London, London, UK; Department of Life Sciences, College of Health and Life Sciences, Brunel University London, London, UK; Department of Clinical Physiology, Tampere University Hospital, and Finnish Cardiovascular Research Center Tampere, Tampere University, Tampere, Finland; Systems Epidemiology, Faculty of Medicine, University of Oulu, Oulu, Finland; Research Unit of Population Health, Faculty of Medicine, University of Oulu, Oulu, Finland; Biocenter Oulu, University of Oulu, Oulu, Finland; Department of Public Health and Welfare, Finnish Institute for Health and Welfare, Helsinki, Finland; Research Centre of Applied and Preventive Cardiovascular Medicine, University of Turku, Turku, Finland; Centre for Population Health Research, University of Turku and Turku University Hospital, Turku, Finland; Department of Clinical Physiology and Nuclear Medicine, Turku University Hospital, Turku, Finland; Department of Clinical Chemistry, Fimlab Laboratories, and Finnish Cardiovascular Research Center Tampere, Tampere University, Tampere, Finland; Systems Epidemiology, Faculty of Medicine, University of Oulu, Oulu, Finland; Research Unit of Population Health, Faculty of Medicine, University of Oulu, Oulu, Finland; Biocenter Oulu, University of Oulu, Oulu, Finland; Systems Epidemiology, Faculty of Medicine, University of Oulu, Oulu, Finland; Research Unit of Population Health, Faculty of Medicine, University of Oulu, Oulu, Finland; Biocenter Oulu, University of Oulu, Oulu, Finland; NMR Metabolomics Laboratory, School of Pharmacy, University of Eastern Finland, Kuopio, Finland; Systems Epidemiology, Faculty of Medicine, University of Oulu, Oulu, Finland; Research Unit of Population Health, Faculty of Medicine, University of Oulu, Oulu, Finland; Biocenter Oulu, University of Oulu, Oulu, Finland; NMR Metabolomics Laboratory, School of Pharmacy, University of Eastern Finland, Kuopio, Finland

**Keywords:** Metabolomics, urine, biomarkers, kidney function, metabolism

## Abstract

**Background:**

Urinary metabolomics has demonstrated considerable potential to assess kidney function and its metabolic corollaries in health and disease. However, applications in epidemiology remain sparse due to technical challenges.

**Methods:**

We added 17 metabolites to an open-access urinary nuclear magnetic resonance metabolomics platform, extending the panel to 61 metabolites (*n* = 994). We also introduced automated quantification for 11 metabolites, extending the panel to 12 metabolites (+creatinine). Epidemiological associations between these 12 metabolites and 49 clinical measures were studied in three independent cohorts (up to 5989 participants). Detailed regression analyses with various confounding factors are presented for body mass index (BMI) and smoking.

**Results:**

Sex-specific population reference concentrations and distributions are provided for 61 urinary metabolites (419 men and 575 women), together with methodological intra-assay metabolite variations as well as the biological intra-individual and epidemiological population variations. For the 12 metabolites, 362 associations were found. These are mostly novel and reflect potential molecular proxies to estimate kidney function, as the associations cannot be simply explained by estimated glomerular filtration rate. Unspecific renal excretion results in leakage of amino acids (and glucose) to urine in all individuals. Seven urinary metabolites associated with smoking, providing questionnaire-independent proxy measures of smoking status in epidemiological studies. Common confounders did not affect metabolite associations with smoking, but insulin had a clear effect on most associations with BMI, including strong effects on 2-hydroxyisobutyrate, valine, alanine, trigonelline and hippurate.

**Conclusions:**

Urinary metabolomics provides new insight on kidney function and related biomarkers on the renal-cardiometabolic system, supporting large-scale applications in epidemiology.

Key Messages

- This work appears as the first comprehensive quantitative urine metabolomics study at an epidemiological scale, and presents 362 associations (most of them novel) between 12 urinary metabolites and 49 clinical and biochemical measures, with replication in three independent population cohorts of up to 5989 participants.
- All individuals have amino acids in the urine. Thus, despite the high efficiency of the amino acid transporters, the large volume of plasma filtered would result in unspecific leakage of amino acids into the urine, similarly to the situation with glucose.
- Seven urinary metabolites associated with smoking, providing questionnaire-independent proxy measures of smoking status in epidemiological studies. Common confounders did not affect these associations.
- Of the 12 urinary metabolites, only dimethylamine and urea did not associate with body mass index (BMI). Insulin had a clear effect on most associations with BMI, including strong effects on 2-hydroxyisobutyrate, valine, alanine, trigonelline and hippurate.
- The novel extensive quantitative urinary metabolomics data and the plethora of associations with clinically relevant measures and outcomes support large-scale epidemiological studies for new insight on kidney function and related disease biomarkers.

## Introduction

A limited number of urinary biomarkers are widely used as diagnostic aids in kidney disease (creatinine and albumin) and diabetes (glucose). The measurement rationale for these is mainly to pinpoint high values that cross pre-set diagnostic limits, for example with the standard urinary glucose test strips with detection limits as high as 5.6 mmol/L.^1^ Quantitative metabolic approaches with large enough numbers of individuals for appropriate epidemiological studies, targeting improved understanding of urinary metabolites in health and as potential biomarkers of disease risk, are almost non-existent.^2–8^ This is at an immense contrast to the situation with various metabolomics and lipidomics approaches already in widespread use in genetics and epidemiology for large-scale quantitative studies of systemic blood biomarkers.^9–15^

Nevertheless, the potential of urinary metabolites in epidemiology and translational medicine has been recognized for quite some time.^2–5^^,^^7^^,^^16^ The molecular content of urine is physiologically connected to the glomerular filtration and molecular reabsorption processes in the kidneys, and reflects multiple key biochemical pathways in relation to cardiometabolic conditions, gut microbial metabolic activities and dietary characteristics. Detailed quantitative data on urinary metabolites may thus provide direct molecular probes to assess kidney function and its corollaries in various metabolic conditions. Towards this far-reaching aim, we have recently developed a basis for an open-access methodology for quantitative high-throughput urinary nuclear magnetic resonance (NMR) metabolomics.^1^^,^^7^^,^^17^

The focus of this work is to extend the population-level quantitative data to 61 urinary metabolites, and to provide their sex-specific reference concentrations and distributions in a population sample of 994 individuals. The first coherent set of automated quantification models for 12 urinary metabolites (+ creatinine) is also presented, together with large-scale assessment of these metabolite concentrations in morning spot urine samples and their associations with wide-ranging clinical data, in three independent population cohorts of up to 5989 participants. This work extends the epidemiological scale of urine metabolomics to a new level, incorporates independent replication and provides a plethora of novel metabolic findings in relation to kidney function, with potential translational relevance.

## Material and methods

### General aspects and data

**Figure 1.**
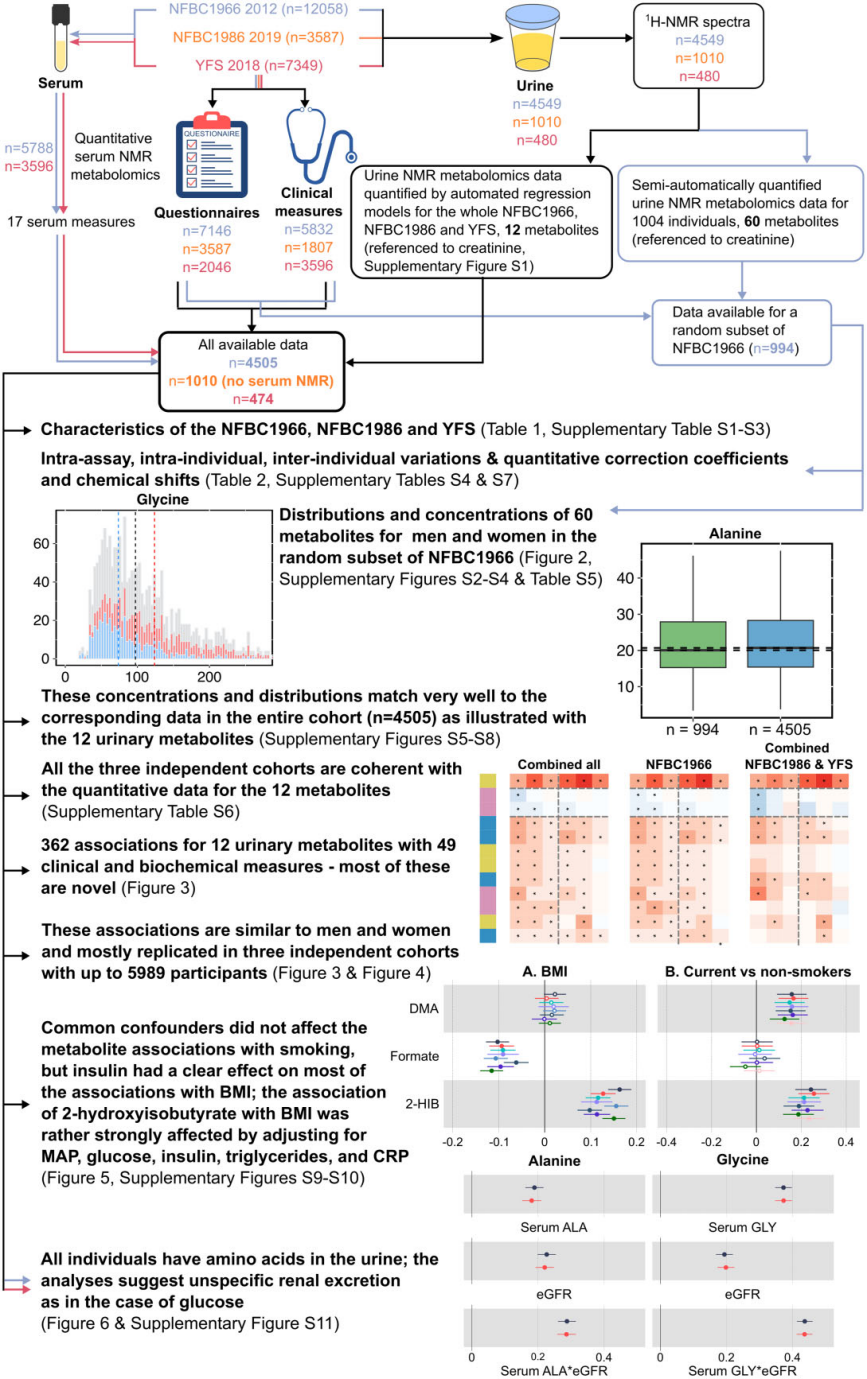
A flowchart illustrating the study design, statistical analyses and key findings. The data from NFBC1966, NFBC1986 and YFS are indicated by colour-coded arrows: blue, orange and red, respectively. The black arrows represent analyses for all the cohorts. NFBC, Northern Finland Birth Cohort; YFS, Cardiovascular Risk in Young Finns Study; NMR, nuclear magnetic resonance; ^1^H-NMR, proton nuclear magnetic resonance; BMI, body mass index; MAP, mean arterial pressure; CRP, C-reactive protein; eGFR, estimated glomerular filtration rate

This work is based on an open-access proton NMR spectroscopy methodology we have recently introduced.^7^ A study outline and a summary of various analyses performed are shown in a schematic form in Figure 1. The characteristics of the three independent population cohorts are given in Table 1. The cohorts are described in more detail in the online Supplementary data together with the detailed list of the 49 clinical and biochemical measures. The sex-specific characteristics for each cohort are given in Supplementary Table S1, available as Supplementary data at *IJE* online (Northern Finland Birth Cohort 1966; NFBC1966, *n* = 4505), Supplementary Table S2, available as Supplementary data at *IJE* online (Northern Finland Birth Cohort 1986; NFBC1986, *n* = 1010)^18^ and Supplementary Table S3, available as Supplementary data at *IJE* online (Cardiovascular Risk in Young Finns Study; YFS, *n* = 474).

**Table 1.** Characteristics of the three independent study populations^a^

Characteristic	NFBC1966	NFBC1986	YFS
Number	4505	1010	474
Age (years)	46.7 (46.2–47.1)	33.7 (33.4–34.1)	50.1 (31.3–68.5)
BMI (kg/m^2^)	26 (23–29)	25 (23–28)	27 (24–30)
Waist-to-hip ratio	0.91 (0.85–0.97)	0.91 (0.87–0.97)	
Body fat (%)	28 (22–35)	26 (20–34)	
Visceral fat area (cm^2^)	99 (76–127)	81 (59–121)	
Total body water (L)	39 (34–47)	39 (33–47)	
Systolic blood pressure (mmHg)	124 (114–135)	111 (103–120)	128 (117–142)
Diastolic blood pressure (mmHg)	84 (77–92)	74 (68–80)	78 (71–85)
Pulse (beats/min)	69 (62–77)	71 (64–79)	69 (62–77)
Fitness score	74 (69–79)	75 (70–80)	
Basal metabolic rate (calories)	1509 (1358–1756)	1520 (1353–1753)	
Grip strength average (kg)	32 (26–45)	35 (30–47)	
Smoking prevalence (%)^b^	17.5	14	18.7
Leucine (mmol/L)	0.08 (0.07–0.10)		0.12 (0.10–0.14)
Isoleucine (mmol/L)	0.05 (0.05–0.07)		0.06 (0.05–0.07)
Valine (mmol/L)	0.20 (0.18–0.23)		0.23 (0.21–0.26)
Alanine (mmol/L)	0.45 (0.41–0.5)		0.37 (0.32–0.43)
Glutamine (mmol/L)	0.57 (0.53–0.61)		0.75 (0.70–0.80)
Glycine (mmol/L)	0.29 (0.26–0.33)		0.26 (0.23–0.32)
Phenylalanine (mmol/L)	0.08 (0.07–0.08)		0.06 (0.05–0.07)
Tyrosine (mmol/L)	0.06 (0.05–0.06)		0.06 (0.06–0.07)
Glycated haemoglobin (%)	5.5 (5.2–5.7)	5.2 (5.0–5.4)	5.5 (5.3–5.8)
Fasting insulin (IU/L)	7.9 (5.4–11.7)		9.2 (5.4–13.4)
Fasting glucose (mmol/L)	5.4 (5.1–5.8)	4.9 (4.7–5.2)	5.4 (5.1–5.8)
Lactate (mmol/L)	1.4 (1.2–1.7)		2.0 (1.7–2.4)
Pyruvate (mmol/L)	0.09 (0.08–0.12)		0.06 (0.05–0.08)
Citrate (mmol/L)	0.12 (0.11–0.13)		0.04 (0.04–0.05)
Glycerol (mmol/L)	0.07 (0.06–0.09)		0.12 (0.09–0.15)
Apolipoprotein B (g/L)	1.02 (0.88–1.2)		0.93 (0.79–1.11)
Total triglycerides (mmol/L)	1.03 (0.76–1.47)	0.78 (0.57–1.09)	1.13 (0.85–1.55)
Apolipoprotein A-I (g/L)	1.7 (1.6–1.9)		1.5 (1.4–1.7)
HDL cholesterol (mmol/L)	1.5 (1.3–1.8)	1.4 (1.2–1.7)	1.3 (1.1–1.6)
Acetoacetate (mmol/L)	0.04 (0.03–0.05)		0.02 (0.01–0.04)
Beta-hydroxybutyrate (mmol/L)	0.12 (0.10–0.16)		0.05 (0.02–0.11)
C-reactive protein (mg/L)	0.82 (0.45–1.65)	0.71 (0.36–1.55)	1.03 (0.53–2.37)
GlycA (mmol/L)	1.4 (1.3–1.5)		0.89 (0.81–0.96)
Haemoglobin (g/L)	141 (132–150)	136 (128–146)	144 (137–152)
Leukocytes (× 10^9^ cells/L)	5.4 (4.5–6.4)		6.1 (5.2–7.1)
Platelets (× 10^9^ cells/L)	247 (215–286)	239 (210–274)	254 (219–292)
Erythrocytes (× 10^12^ cells/L)	4.7 (4.4–4.9)	4.6 (4.3–4.9)	4.8 (4.5–5.1)
Bilirubin (µmol/L)	11 (9–15)	12 (9–16)	
Alkaline phosphatase (U/L)	61 (51–73)	55 (45–66)	
Alanine aminotransferase (U/L)	25 (18–36)	21 (16–31)	22 (15–31)
Gamma-glutamyl transferase (U/L)	23 (15–38)	15 (11–24)	24 (17–38)
Uric acid (µmol/L)	297 (249–353)	303 (252–356)	
Creatinine (µmol/L)	67 (59–75)	65 (58–74)	76 (67–87)
eGFR (mL/min/1.73m^2^)	104 (95–107)	115 (107–118)	87 (75–100)
FINRISK	0.57 (0.20–1.66)	0.10 (0.04–0.32)	1.19 (0.2–6.62)
CKD Nelson risk	1.4 (1.0–2.4)	0.29 (0.23–0.47)	6.6 (1.2–31.7)
CKD O’Seaghdha risk	0.76 (0.76–1.61)	0.24 (0.23–0.25)	3.65 (0.50–20.89)
CKD Chien risk	6.1 (4.7–8.4)	1.8 (1.4–2.3)	10.2 (2.7–40)

BMI, body mass index; CKD, chronic kidney disease; eGFR, estimated glomerular filtration rate; FINRISK, a large Finnish population survey of risk factors for chronic, noncommunicable diseases; GlycA, glycoprotein acetyls; HDL, high-density lipoprotein; NFBC, Northern Finland Birth Cohort; YFS, Cardiovascular Risk in Young Finns Study.

a Values are median (interquartile range).

b The number of current smokers/the total number of cohort participants.

**Figure 2.**
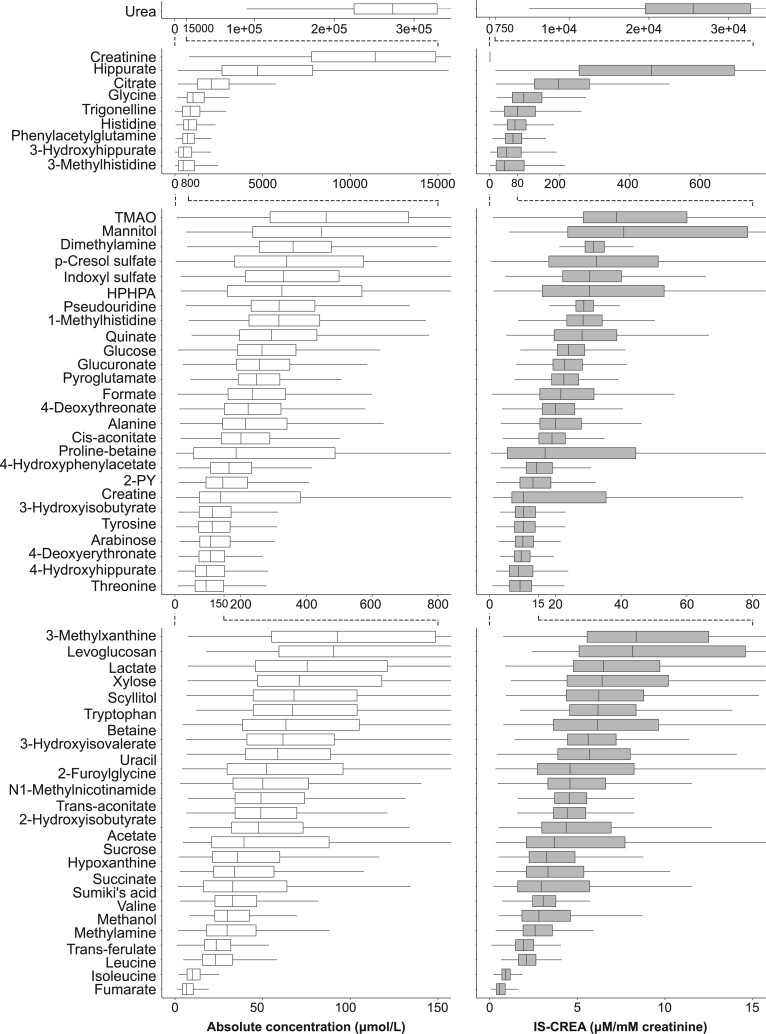
Absolute (left) and creatinine-referenced (right) concentrations of 61 quantified urinary metabolites in a random subset (*n *= 994) of morning spot urine samples in the Northern Finland Birth Cohort 1966. The metabolites are presented in the descending order of median absolute concentrations. Several different scales are used for the x-axes to provide a clear visualization for the large concentration ranges. TMAO, trimethylamine N-oxide; HPHPA, 3-(3-hydroxyphenyl)-3-hydroxypropanoate; 2-PY, N1-methyl-2-pyridone-5-carboxamide; IS-CREA, use the creatinine concentration as the internal standard

In addition to the novel data on clinical and biochemical associations, we extend the methodology here from the previously quantified 43 urinary metabolites (+creatinine) to 60 metabolites (+creatinine), and present the methodological intra-assay metabolite coefficients of variation (CV %s) as well as 30-day consecutive intra-individual and inter-individual population variation for the added 17 metabolites (Table 2). The corresponding previously published information^7^ for the 43 metabolites is given for the convenience of the readers in Supplementary Table S4, available as Supplementary data at *IJE* online (together with the data for the added 17 metabolites). These analyses were done in a random subset of 994 participants in the NFBC1966, and the distributions for all these metabolites are illustrated in Figure 2.

**Table 2.** Intra-assay, intra-individual and inter-individual variation of 17 quantified urine metabolites (introduced in this work)^f^

Metabolite	Intra-assay	Intra-individual	Inter-individual
	**CV (%)** ^a^^,^^b^	**CV (%)** ^a^^,^^c^	**CV (%)** ^a^^,^^d^
Amino acids			
Leucine	6.34	26.48	58.25
Metabolism of amino acids			
Betaine	3.23	43.96	176.13
Phenylacetylglutamine	3.12	31.90	54.00
Pyroglutamate	4.40	17.64	27.64
Carbohydrate metabolism			
Fumarate	—^e^	176.23	218.72
Succinate	13.36	32.08	179.91
Mannitol	—^e^	164.85	222.25
Caffeine metabolism			
3-methylxanthine	5.02	174.27	84.08
Microbial metabolism			
Methanol	1.91	60.33	114.21
Dietary metabolites			
1-methylhistidine	2.04	21.15	31.26
Levoglucosan	1.52	304.04	190.10
Proline-betaine	2.71	132.03	139.93
Quinate	3.51	262.56	81.67
Scyllitol	1.19	22.05	57.91
Trans-ferulate	4.71	31.12	101.66
Miscellaneous			
4-deoxyerythronate	1.59	18.15	38.46
4-deoxythreonate	1.67	30.42	38.60

Identical data for 43 metabolites from a previous publication are shown for the convenience of readers in Supplementary Table S4 (available as Supplementary data at *IJE* online).

CV, coefficient of variation.

a Concentrations are scaled to the concentration of creatinine; CV (%) = (standard deviation/average) * 100%.

b One urine sample prepared and analysed as 10 replicates; reflects the entire quantitative process, i.e. including all the sample preparation steps, nuclear magnetic resonance experimentation and mathematical quantification protocols.

c A 30-day consecutive urine collection, averaged over three different volunteers.

d In 1003 different individuals from the Northern Finland Birth Cohort 1966.

e Concentration of the metabolite below the detection limit in this urine sample.

In this work we present automated quantification models for 12 urinary metabolites (+ creatinine) (Supplementary Figure S1, available as Supplementary data at *IJE* online). These models enabled coherent analyses of urinary metabolite concentrations in three independent population cohorts (up to 5989 participants), and replicated association analyses with wide-ranging clinical data. A representative set of 49 clinical and biochemical measures, 17 of which are based on serum NMR metabolomics,^7^^,^^10^^,^^19^^,^^20^ were chosen for the association analyses. For the details of the urine sample preparation and the NMR spectroscopy experimentation, we refer to earlier open-access publications.^7^^,^^17^

### Metabolite quantification and analytical issues

The 61 urinary metabolites (Figure 2) were quantified from the NMR spectra with a semi-automated methodology using sophisticated constrained total line shape (CTLS) fitting analysis.^7^^,^^21^^,^^22^ These analyses are tedious and time consuming, and are not feasible in large-scale epidemiology applications. This is the very reason why we started to develop an automated regression analysis approach for urine NMR metabolomics,^7^ as this approach has proved superior in the case of quantitative serum NMR metabolomics, with current data available for a plethora of various epidemiological and genetic applications and spanning to 1.5 million samples and counting.^10^^,^^13^^,^^14^^,^^23^^,^^24^

Additional boxplots (Supplementary Figure S2, available as Supplementary data at *IJE* online) and histograms (Supplementary Figures S3 and S4, available as Supplementary data at *IJE* online) are available in the online Supplementary data for the 61 urinary metabolites. Importantly, the concentration and distribution data for the random subset match very well to the corresponding data in the entire cohort of 4505 participants (Supplementary Figures S5–S8, available as Supplementary data at *IJE* online). The numerical data for the urinary metabolite concentrations in men and women for the NFBC1966 subset (60 metabolites + creatinine) can be found in Supplementary Table S5 (available as Supplementary data at *IJE* online), and for all the three cohorts (12 metabolites + creatinine) in Supplementary Table S6 (available as Supplementary data at *IJE* online).

Results from only two automated regression models, glucose and creatinine, have been published previously.^1^^,^^7^ In this work we add 11 automated quantification models, namely 2-hydroxyisobutyrate, valine, alanine, pseudouridine, dimethylamine, glycine, citrate, urea, formate, trigonelline and hippurate. Assessments of the automated regression models for these metabolites are available in Supplementary Figure S1 (available as Supplementary data at *IJE* online) together with the population distributions for ∼4500 individuals in NFBC1966.

In this study we also determined the correction coefficients for each quantified metabolite, to lead to the true absolute metabolite concentrations (Supplementary Table S7, available as Supplementary data at *IJE* online).

### Statistical analyses

Urinary metabolite concentrations normalized to urinary creatinine concentration were used in all analyses, but absolute concentrations are also presented (Figure 2). Referencing to creatinine has been a long-term standard choice in urine NMR metabolomics, but we have also recently comprehensively studied the effects of various normalization methods, also supporting the use of creatinine referencing in epidemiological studies.^17^

**Figure 3.**
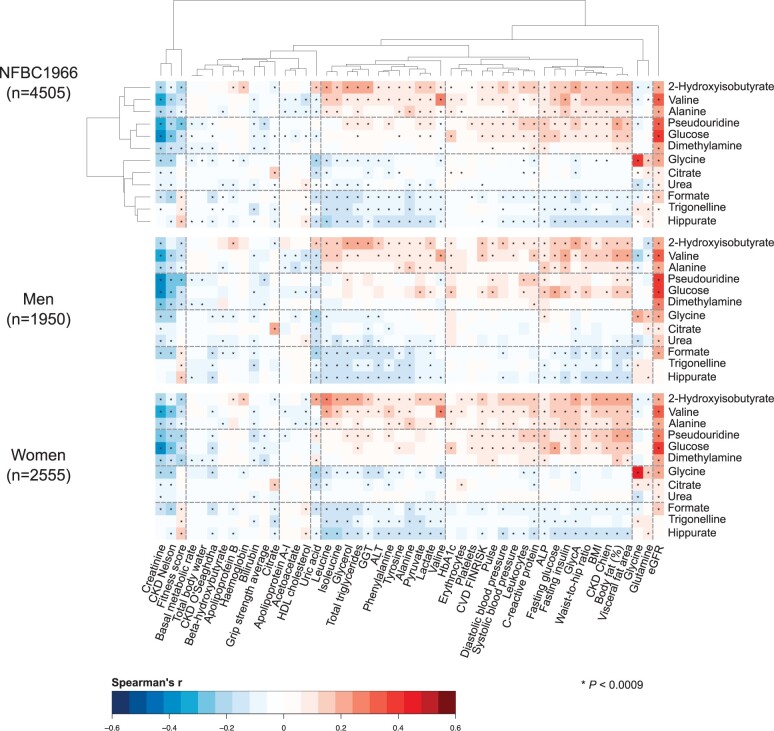
The associations between the 12 automatically quantified urinary metabolites (referenced to urinary creatinine) and 49 customary clinical and biochemical measures as indicated by Spearman’s rank correlations (adjusted for sex) for the entire Northern Finland Birth Cohort 1966 (*n =* 4505) as well as for men (*n* = 1950) and women (*n* = 2555). The two-dimensional hierarchical clustering is based on the results for the entire cohort, and the resulting ordering is preserved in all the following heat maps. Four three-metabolite clusters were rendered that reflect the clinical and biochemical associations of the urinary metabolites. *P*-value <0.0009 is marked with an asterisk in the map to indicate a multiple testing corrected association. ALP, alkaline phosphatase; ALT, alanine aminotransferase; GGT, gamma-glutamyl transferase; eGFR, estimated glomerular filtration rate; CKD, chronic kidney disease; HbA1c, glycated haemoglobin; BMI, body mass index; GlycA, glycoprotein acetyls; FINRISK, a large Finnish population survey of risk factors for chronic, noncommunicable diseases

**Figure 4.**
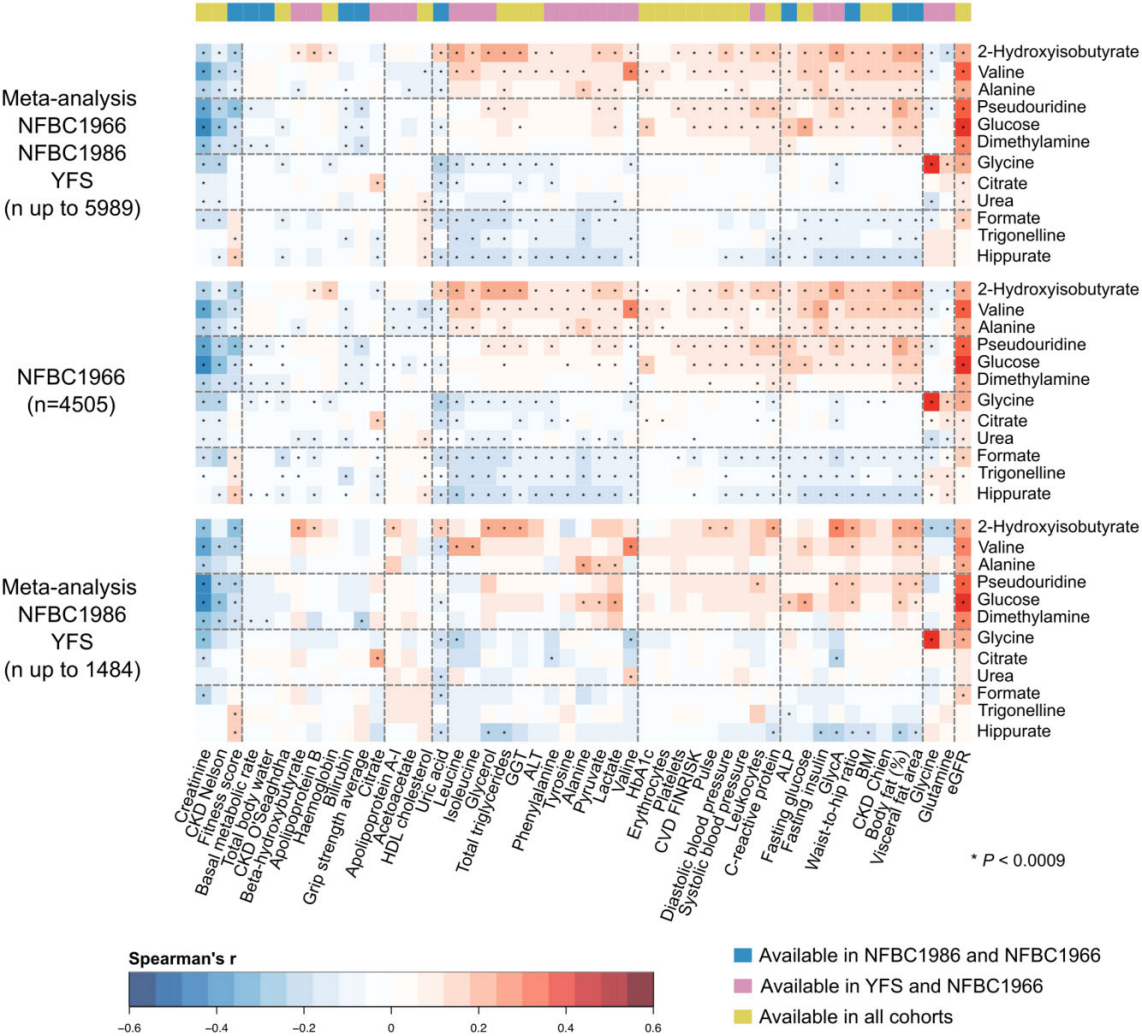
Meta-analyses of the associations (Spearman’s rank correlations adjusted for sex) between the 12 automatically quantified urinary metabolites (referenced to urinary creatinine) and 49 customary clinical and biochemical measures, to illustrate the replication of the findings in all the three independent population cohorts. The uppermost heat map shows the full meta-analyses for all the available data (*n* up to 5989). The heat map in the middle is for the entire NFBC1966 (the same heat map as in Figure 3, to facilitate visual comparison). The lowermost heat map shows the meta-analysis for NFBC1986 and YFS (*n* up to 1484). The heat maps are presented in the same order of metabolites and clusters as in Figure 3. The colour key on the top of the figure represents the availability of clinical and biochemical measures in the three cohorts. There were 20 measures available in all three cohorts (green), 19 measures available only in NFBC1966 and YFS (pink) and 10 measures available only in NFBC1966 and NFBC1986 (blue). *P*-value <0.0009 is marked with an asterisk in the map to indicate a multiple testing corrected association. ALP, alkaline phosphatase; ALT, alanine aminotransferase; GGT, gamma-glutamyl transferase; eGFR, estimated glomerular filtration rate; CKD, chronic kidney disease; HbA1c, glycated haemoglobin; BMI, body mass index; GlycA, glycoprotein acetyls; FINRISK, a large Finnish population survey of risk factors for chronic, noncommunicable diseases; NFBC, Northern Finland Birth Cohort; YFS, Cardiovascular Risk in Young Finns Study

Partial rank correlations (adjusted for sex in all the cohorts and in addition for age in YFS) were used to illustrate the associations between the 12 automatically quantified urinary metabolites and the 49 clinical and biochemical measures. The results are shown in colour-coded heat maps in Figure 3 for the biggest individual cohort, NFBC1966, with separate maps for the entire cohort (*n* = 4505) as well as for men (*n* = 1950) and women (*n* = 2555). The two-dimensional hierarchical clustering is based on the results for the entire cohort, and the resulting ordering is preserved in all the following heat maps. The chemical taxonomy of the metabolites can be seen in Table 2.^17^ Replication of the associations is illustrated in Figure 4. In all, 56 principal components explained over 99% of variation in the 60 creatinine-referenced urinary metabolite concentrations and the 49 clinical and biochemical measures in NFBC1966. Therefore, we used a multiple comparison corrected *P*-value threshold of 0.0009 to suggest evidence in favour of an association (0.05/56 via the Bonferroni method; *P *<0.0009 is denoted in the figures with an asterisk).

**Figure 5.**
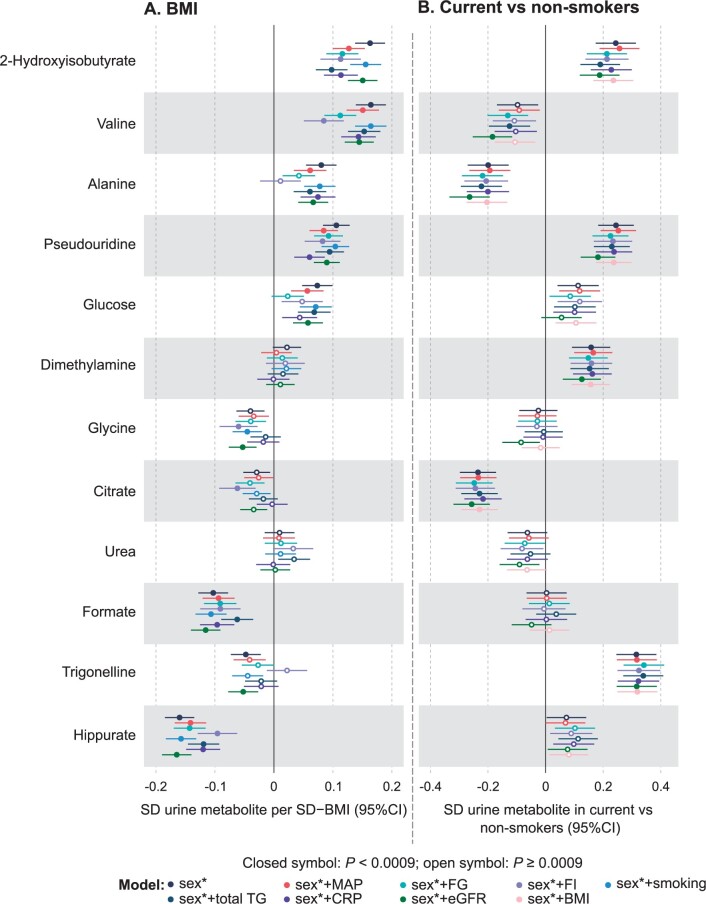
Meta-analyses of the regression models for body mass index (A) and smoking (B) with the 12 automatically quantified urinary metabolites (referenced to creatinine). The effects of sex (black), sex + MAP (red), sex + fasting glucose (cyan), sex + fasting insulin (lila), sex + smoking (light blue, applied to the BMI models only), sex + total triglycerides (blue), sex + CRP (violet), sex + eGFR (green) and sex + BMI (blush, applied to the smoking models only) were examined; asterisk indicates that age was also adjusted for YFS. The smoking data for the cohorts are: NFBC1966, 750 current and 3544 non-smokers; NFBC1986, 115 current and 706 non-smokers; and YFS, 85 current and 370 non-smokers. MAP, mean arterial pressure; CRP, C-reactive protein; eGFR, estimated glomerular filtration rate; YFS, Cardiovascular Risk in Young Finns Study; NFBC, Northern Finland Birth Cohort

Associations between the urinary metabolites and body mass index (BMI), as well as smoking history (current smokers vs non-smokers), were analysed via linear regression analyses adjusted for sex in all the cohorts and in addition for age in YFS. Extreme metabolite levels (metabolites >third quartile + 8 * interquartile range) were truncated to the values of the upper bound, and the metabolite concentrations were log-transformed. The truncation was done as a precaution, since the extreme values are rare (Supplementary Table S8, available as Supplementary data at *IJE* online), represent real metabolite concentrations (not artefacts) and did not have strong effects on the associations. All measures were scaled to standard deviation (SD) units (by subtracting the mean and dividing by the standard deviation). Association magnitudes are reported in SD units to ease the comparison across multiple measures with different initial units and scales. All models were further individually adjusted for mean arterial pressure, fasting glucose, fasting insulin, smoking history (BMI analysis only), total triglycerides, C-reactive protein (CRP), estimated glomerular filtration rate (eGFR) and BMI (smoking analysis only). Individual adjustments for the confounders were performed to understand the relations and potential mediation of the urinary metabolite associations with the clinical and biochemical measures and outcomes. All analyses were done separately in the individual cohorts (Supplementary Figure S9, available as Supplementary data at *IJE* online, for BMI and Supplementary Figure S10, available as Supplementary data at *IJE* online, for smoking) and then meta-analysed (Figure 5A for BMI and Figure 5B for smoking).

**Figure 6:**
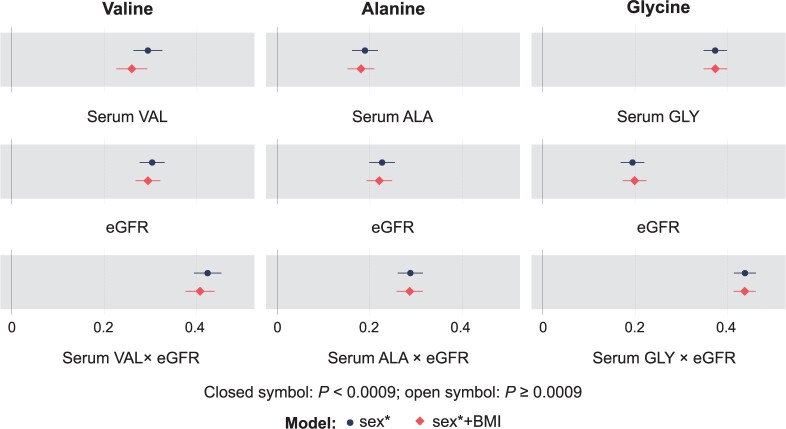
Meta-analyses of the regression models for the three automatically quantified urinary amino acids (valine, alanine and glycine) concentrations (referenced to creatinine) and their corresponding serum concentrations, eGFR, and the multiplication of the serum concentration and eGFR in Northern Finland Birth Cohort 1966 (*n* = 4505) and Cardiovascular Risk in Young Finns Study (*n* = 474). The effects of sex (black circle) and sex + BMI (red diamond) were examined; asterisk indicates that age was also adjusted for in YFS. eGFR, estimated glomerular filtration rate; YFS, Cardiovascular Risk in Young Finns Study

Three regression analyses for each of the three urinary amino acid concentrations (valine, alanine and glycine) were also performed (as in our previous work for glucose^1^), namely with their corresponding serum concentrations, eGFR and the multiplication of serum concentration and eGFR. The models were further adjusted for BMI. These data were available for NFBC1966 and YFS, so the analyses were first done separately in these two cohorts (Supplementary Figure S11, available as Supplementary data at *IJE* online) and then meta-analysed (Figure 6).

## Results

### Metabolite distributions, abundance and sex differences

Figure 2 illustrates the absolute and urine creatinine-referenced concentrations of 61 quantified urinary metabolites in 994 morning spot urine samples. The set of 61 metabolites presented here represents all the most abundant signals in the urine NMR spectra. Urea is by far the most abundant metabolite, with a median absolute concentration greater than 200 mM. Creatinine is also an abundant metabolite, with a median absolute concentration greater than 10 mM. Hippurate and citrate are present in median absolute concentrations greater than 1 mM. The population variation of the metabolites is substantial (Figure 2, Table 2 and Supplementary Table S4, available as Supplementary data at *IJE* online) and many of the metabolite distributions are positively skewed (Supplementary Figures S2–S4, available as Supplementary data at *IJE* online). In creatinine-referenced data, many metabolites are slightly more abundant in women than in men, but the concentration differences are small (Supplementary Figures S2–S4 and Supplementary Table S5, available as Supplementary data at *IJE* online).

The methodological intra-assay metabolite CV%s for the new 17 metabolites are similar to those for the earlier 43 metabolites, i.e. mostly less than 5%, indicating high consistency and accuracy of urine NMR spectroscopy per se. Also their 30-day consecutive intra-individual and inter-individual population variations follow the same overall pattern as reported earlier,^7^ with rather large intra-individual and typically even larger inter-individual variation (Table 2 and Supplementary Table S4, available as Supplementary data at *IJE* online). Figure 2, together with the detailed information in Supplementary Table S5 (available as Supplementary data at *IJE* online), provide valuable reference concentrations for key urinary metabolites at a population level.

### Association clusters

Figure 3 illustrates a colour-coded heat map of associations between 12 urinary metabolites (referenced to urinary creatinine) and 49 customary clinical and biochemical measures (detailed descriptions are available in online Supplementary data). The associations depicted are mostly novel, since quantitative data on urinary metabolites at an epidemiological scale are scarce. Even though the associations are overall rather weak, 362 associations were detected which fulfilled the statistical multiple comparison corrected *P*-value threshold of 0.0009. To facilitate the metabolic interpretation of the results, a two-dimensional hierarchical clustering of the heat map was done based on the sex-adjusted associations in the entire NFBC1966 cohort (*n* = 4505). Four three-metabolite clusters were rendered which reflect the clinical and biochemical associations of the 12 urinary metabolites.

The strongest association appears between urinary and serum glycine, and the association between urinary valine and serum valine is rather strong. The estimated glomerular filtration rate (eGFR) associates positively with all the urinary metabolite clusters, the strongest associations being with glucose, pseudouridine and valine. These associations are, as expected, mirrored by negative associations with serum creatinine (which is used in the estimation of glomerular filtration rate). The two uppermost metabolite clusters in Figure 3 (the first one consisting of 2-hydroxyisobutyrate, valine and alanine and the second one of pseudouridine, glucose and dimethylamine) behave generally similarly regarding their associations. They are overall positive with over 60% of the clinical and biochemical measures, including eGFR as noted above, multiple serum amino acids, serum triglycerides, glycaemic traits, lactate, pyruvate, inflammation (CRP and GlycA), liver function markers [alkaline phosphatase (ALP), alanine aminotransferase (ALT) and gamma-glutamyl transferase (GGT)], various obesity measures, blood pressure and the FINRISK cardiovascular disease (CVD) and the Chien chronic kidney disease (CKD) risk scores.

The associations of the two downmost metabolite clusters in Figure 3 (the first one consisting of glycine, citrate and urea and the second one of formate, trigonelline and hippurate) also behave generally similarly in their associations, though the former of these has the lowest number of associations with the clinical and biochemical measures. Their associations are, in general, negative for most of the above-mentioned positive associations of the two uppermost metabolite clusters. However, the associations are somewhat reversed for serum glycine and glutamine, with weak negative associations with the two uppermost metabolite clusters and mixed negative and positive associations with the two downmost clusters. In addition to serum creatinine, serum citrate, bilirubin and the CKD Nelson, as well as the O'Seaghdha risk scores, tend to associate negatively with all the urinary metabolite clusters and metabolites. Apolipoprotein B associates positively with the uppermost urinary metabolite cluster and negatively with the downmost cluster. The associations for high-density lipoprotein (HDL) cholesterol are opposite to those of apolipoprotein B. The fitness score associates negatively with the two uppermost metabolite clusters and positively with the lowermost cluster.

The associations of the urinary metabolite clusters and of the individual urinary metabolites with the clinical and biochemical measures are similar for men and women (Figure 3). In addition, the associations appear coherent between three independent cohorts, as illustrated in Figure 4. The NFBC1986 (*n* = 1010) and YFS (*n* = 474) had fewer urine samples available than the NFBC1966 (*n *= 4505), and thus only the most prominent associations reach the multiple comparison corrected *P*-value threshold of 0.0009. Nevertheless, the entire association pattern matches excellently with the one for NFBC1966.

### Metabolite associations with BMI

The associations of the 12 urinary metabolites, referenced to creatinine, with BMI are shown in Figure 5A, meta-analysed for the three independent cohorts (*n* up to 5989). The results for the individual cohorts are shown in Supplementary Figure S9 (available as Supplementary data at *IJE* online). Only urea and dimethylamine did not associate with BMI. Fasting insulin had a strong effect on the associations of several urinary metabolites with BMI. Apart from the effects of fasting insulin, the other adjustments in the regression models had overall very little, if any, effects on the associations. However, adjusting for fasting glucose had a similar but less pronounced effect on valine and alanine as fasting insulin. Adjusting for fasting glucose also had (an expected) strong effect on diluting the association of urinary glucose with BMI. The association of 2-hydroxyisobutyrate was rather strongly affected by adjusting for mean arterial pressure, fasting glucose, fasting insulin, total triglycerides and CRP.

### Metabolite associations with smoking

The associations of the 12 urinary metabolites referenced to creatinine with smoking are shown in Figure 5B, meta-analysed for the three independent cohorts (*n* up to 5989). The results for the individual cohorts are shown in Supplementary Figure S10 (available as Supplementary data at *IJE* online). Seven metabolites associated with smoking at the multiple testing corrected *P*-value threshold <0.0009, namely 2-hydroxyisobutyrate, valine, alanine, pseudouridine, dimethylamine, citrate and trigonelline. The various adjustments had very little, if any, effect on the urinary metabolite associations with smoking.

### Unspecific renal excretion of amino acids

The 12 quantified urinary metabolites include three amino acids, valine, alanine and glycine. Figure 6 illustrates how these urinary amino acid concentrations associate with corresponding serum concentrations and eGFR, and that these associations are strengthened for the multiplication of the serum concentration and eGFR. The results shown are meta-analysed for the NFBC1966 and YFS, for which the serum amino acid data were available. Results for the individual cohorts are given in Supplementary Figure S11 (available as Supplementary data at *IJE* online).

## Discussion

Novel quantitative data are presented here for 60 urinary metabolites (+ creatinine) in a 994-individual subset of the NFBC1966 cohort. This random subset is representative of the entire cohort of 4505 participants (Supplementary Figures S5–S8, available as Supplementary data at *IJE* online) and thus the quantitative data provide valuable reference concentrations for key urinary metabolites at a population level for men and women (Figure 2, Supplementary Figures S2–S4 and Supplementary Table S5, available as Supplementary data at *IJE* online). The concentration differences between males and females are small, but the creatinine-referenced values tend to be slightly higher for women. On average, women have lower muscle mass, leading to lower concentrations of circulating creatinine and thus lower amounts of excreted creatinine into the urine. Whereas in random urine samples absolute metabolite concentrations would not be relevant, in the case of morning spot urine samples, as here and as reflected by Figure 2, the biological variability is to some extent reduced due to the corresponding times and conditions in which the urine has been accumulating in all the participants, i.e. overnight in mostly a fasting physiological state.^25^

The first coherent set of automated quantification models for 12 urinary metabolites (+ creatinine) is also presented in this work (Supplementary Figure S1, available as Supplementary data at *IJE* online). Application of these models made it feasible to analyse these metabolite concentrations in urine samples for almost 6000 people in three independent population cohorts, and to study their associations with a comprehensive set of 49 clinical and biochemical measures. Hierarchical clustering of these results revealed four three-metabolite clusters that comprehensively summarized their association patterns (Figure 3 and Figure 4). Most of the detected associations are novel, since no epidemiological studies have been carried out combining a comprehensive quantitative metabolomics approach in urine samples with an extensive set of attached clinical and biochemical data. Since urinary metabolites overall correlate weakly with systemic metabolic measures,^7^ urine samples are a potential source of unique metabolic information. In addition, quantitative data on specific urinary metabolites provide a direct individual measurement of kidney function and can potentially alleviate generalized approximations in estimated glomerular filtration rate, a known marker of ageing and cardiometabolic diseases.^26^^,^^27^

We noted recently that glucose in the urine is a normal phenomenon (though some renal physiology textbooks still claim otherwise) and that typical absolute glucose concentrations in urine are between 0.1 and 0.5 mmol/L.^1^ This phenomenon is likely a reflection of unspecific renal excretion of glucose also at low concentration ranges of circulating glucose.^1^^,^^28^ Amino acids—as well as glucose—are indispensable in human metabolism, and thus basically all amino acids filtered by the kidneys are also reabsorbed into the circulation (or used in the kidneys) via a set of specific amino acid transporters.^29^^,^^30^ The population distributions for all the nine quantified amino acids in the urine samples (Figure 2; and Supplementary Figures S2–S4 and Supplementary Table S5, available as Supplementary data at *IJE* online) are similar and resemble those of glucose.^1^ In fact, similarly to glucose, all individuals have amino acids in the urine. Thus, despite the high efficiency of the amino acid transporters, the large volume of plasma filtered would result in some unspecific leakage of amino acids into the urine.^28^^,^^29^ As in the case of glucose, the unspecific leakage is suggested for the amino acids by the rather strong correlations between their serum and urine concentrations (Figure 3 and Figure 4) as well as by the additional contribution of the eGFR to the urinary amino acid concentration (Figure 6). Since quantitative metabolic studies of urine samples at the population level are scarce, we know very little about these types of unspecific molecular processes in the kidneys and their potential role as population-level biomarkers for kidney function and/or disease risk.^1^

The multiple associations between urinary metabolites and various clinical and biochemical measures suggest that urine metabolites may well have general value as population-level health and disease biomarkers. For example, the urinary branched-chain amino acid (BCAA) valine associates positively to various obesity markers (e.g. BMI and waist-to-hip ratio, as well as body and visceral fat), clinical diabetes indicators and risk factors [glycated haemoglobin (HbA1c), fasting glucose, fasting insulin, and serum BCAAs valine, leucine and isoleucine), systemic inflammation (CRP and GlycA), serum triglycerides, blood pressure, liver function (ALP, ALT and GGT), and CKD as well as CVD risk (Figure 3). The direction of association is reversed to overall fitness and HDL-related measures. These findings are in accordance with findings related to circulating valine, and in general with serum BCAAs, concentrations. Apart from adding confirmatory data to systemic metabolic findings, a key aspiration in the case of urinary metabolites would be that, if added into the systemic metabolic risk assessment, they might be able to bring in additional information, directly reflecting kidney function. This is supported by our previous findings that urine and serum metabolites generally correlate weakly.^7^ Furthermore, in the regression models of smoking with the urinary metabolite concentrations, adjusting for various key systemic measures (e.g. CRP, blood pressure, BMI, fasting insulin) had very minor or no effects on the associated metabolites 2-hydroxyisobutyrate, valine, alanine, pseudouridine, dimethylamine, citrate and trigonelline (Figure 5B; and Supplementary Figure S10, available as Supplementary data at *IJE* online). This conclusion also applies to adjustments with eGFR, suggesting that the associations of urinary metabolites are typically such that they cannot be simply explained by a standard clinical estimate of kidney function. A similar conclusion regarding eGFR is valid for the urinary metabolite associations with BMI. However, contrary to the overall minor effects due to the adjustments, insulin had a clear effect on most of the associations with BMI (Figure 5A). These findings are in line with recent longitudinal finding on systemic metabolic ageing trends and obesity.^31^^,^^32^

The positive associations of urinary 2-hydroxyisobutyrate with BMI appear most broadly affected by the adjustments, including those for mean arterial pressure, fasting glucose, fasting insulin, total triglycerides and CRP; 2-hydroxyisobutyrate associated positively also with smoking, but none of the adjustments had a clear effect on the association. In general, the associations of 2-hydroxyisobutyrate are very similar to those of valine (Figure 3 and Figure 4), except that urinary valine associates negatively to smoking (Figure 5B). Potentially originating from gut microbial valine degradation, 2-hydroxyisobutyrate is a tertiary alcohol.^4^^,^^33^^,^^34^ Epidemiological data on this metabolite are almost completely lacking,^35^ except its urinary concentration has also previously been associated with BMI.^4^ In addition, a recent study in individuals with type 1 diabetes found urinary 2-hydroxyisobutyrate positively associated with the progression of diabetic nephropathy.^8^ All these finding suggest urinary 2-hydroxyisobutyrate concentrations being linked with insulin resistance.^36^^,^^37^ Some other associations have also been reported, e.g. 2-hydroxyisobutyrate being part of a ‘peculiar obese urinary metabotype’^38^ and associating with various issues of pregnancy.^39^^,^^40^ However, all these studies have applied orthogonal projections to latent structures discriminant analysis (OPLS-DA) supervised analyses that are well-known to lead to spurious findings, particularly when a lot of spectral data points are used as the basis for the classifications in very small datasets.^9^^,^^41–46^

The results discussed above in relation to 2-hydroxyisobutyrate demonstrate how urinary metabolomics can provide substantial scientific novelty. First, because comprehensive quantitative data on urinary metabolites from large-scale epidemiological studies are scarce, and second, because urine as a waste biofluid—tightly connected to the kidney function—provides a metabolic view that is interdependent with and complementing systemic metabolism. In addition, quantitative metabolite data are indispensable to avoid the common limitations of multivariate metabolomics applications (typically the use of OPLS-DA) that result in spurious findings, due to overtraining of classification models with high numbers of variables (usually spectral data points) with very small numbers of individuals.^9^^,^^20^^,^^41^^,^^45–47^ Quantitative metabolite data (identical to data from standard clinical chemistry analyses) also provide easy means for confounding adjustments as applied in this work^48^ and replication of the findings, in this case done in up to three independent cohorts. These are essential elements allowing triangulation^49^ and leading to scientific reliability.^50^

Even though urinary metabolites intrinsically disclose what we eat and drink, the search for discriminatory molecular signals for individual nutrients or even dietary patterns has proved to be futile, ^41^ though some systemic metabolomics studies have been able to link multi-metabolic profiles of individuals with their habitual diets, for example, a preponderance of ‘fruits and vegetables’ or ‘junk food’.^51^ These types of questionnaire-independent ways of assessing true food consumption would be valuable in epidemiological studies. This applies also for explicit gauging of smoking. We showed here that seven metabolites (Figure 5B; and Supplementary Figure S10, available as Supplementary data at *IJE* online) associated with smoking (current vs non-smokers). These findings might prove valuable in large epidemiological studies as a questionnaire-independent assessment of smoking status.

## Conclusion

It is fundamental to keep in mind with these observational epidemiological results that they cannot apprise of any mechanisms. In addition, we did not have direct measures of organ function available, which limits our ability to assess the potential clinical utility of the new metabolites compared with established biomarkers. However, this work gives one of the first demonstrations of the rich and diverse association pattern of urinary metabolites, with multiple descriptors of the renal-cardiometabolic system. Many associations of urinary metabolites with clinical outcomes appear independent of key systemic metabolic regulators, thus suggesting that quantitative urinary metabolomics may inherently convey rather specific information on tubular filtration and reabsorption, as well as on kidney-specific molecular interactions. Keeping in mind that urine is a waste product, a coherent metabolic association pattern in three independent population cohorts is an intriguing result per se. These large-scale results also point towards a very high analytical reliability of the new quantitative methodology, as we noted earlier, based on biologically relevant genetic associations with only a few hundred individuals.^7^ The presented extensive data and results give a dependable rationale to extend quantitative urinary metabolomics to large-scale epidemiological studies for new insight on kidney function and related metabolic disease biomarkers. All the three population cohorts studied in this work are from Finland. Hence, replication of the findings in other ethnicities and geographical locations would be valuable and also of high scientific interest, due to potential societal and environmental effects on urinary metabolite profiles and their clinical associations.

## Ethics approval

The Northern Finland Birth Cohort 1966 and 1986 were approved by the Northern Ostrobothnia Hospital District, Finland. The Cardiovascular Risk in Young Finns Study was approved by the five universities with medical schools in Finland that were involved in the study (Turku, Helsinki, Tampere, Kuopio and Oulu). All participants gave written informed consent.

## Supplementary Material

dyad162_Supplementary_Data

## Data Availability

The datasets used in the current study are available from the cohorts through the application process for researchers who meet the criteria for access to confidential data: [https://www.oulu.fi/nfbc/] and [http://youngfinnsstudy.utu.fi]. Regarding the YFS data, the Ethics Committee has concluded that under applicable law, the data from this study cannot be stored in public repositories or otherwise made publicly available. The data controller may permit access on case-by-case basis for scientific research, not however to individual participant-level data but to aggregated statistical data, which cannot be traced back to the individual participants' data.
