# Supplementary material for: Clinical and biochemical associations of urinary metabolites: quantitative epidemiological approach on renal-cardiometabolic biomarkers
Source: Int J Epidemiol. 2023 Nov 29;53(1):dyad162. doi: 10.1093/ije/dyad162 (PMC10859141; doi:10.1093/ije/dyad162)
Supplement: dyad162_Supplementary_Data [file dyad162_supplementary_data.pdf]

Data Supplement

Clinical and biochemical associations of urinary metabolites: Quantitative epidemiological approach on renal-cardiometabolic biomarkers

Tianqi Li, Andrei Ihanus, Pauli Ohukainen, Marjo-Riitta Järvelin, Mika Kähönen, Johannes Kettunen, Olli T. Raitakari, Terho Lehtimäki, Ville-Petteri Mäkinen, Tuulia Tynkkynen & Mika Ala-Korpela

CONTENTS

Northern Finland Birth Cohorts 1966 and 1986

Cardiovascular Risk in Young Finns Study

Clinical and biochemical measures

Supplementary tables

Table S1. Sex specific characteristics of the Northern Finland Birth Cohort 1966.

Table S2. Sex specific characteristics of the Northern Finland Birth Cohort 1986.

Table S3. Sex specific characteristics of the Cardiovascular Risk in Young Finns Study.

Table S4. Intra-assay variation as well as intra-individual and inter-individual variation of quantified urine metabolites.

Table S5. Concentrations for 61 urinary metabolites in men and women in the random subset of Northern Finland Birth Cohort 1966 (n=994).

Table S6. Concentrations for 13 urinary metabolites in Northern Finland Birth Cohort (NFBC) 1966, NFBC1986 and Cardiovascular Risk in Young Finns Study.

Table S7. Correction coefficients and chemical shifts of the quantified urine metabolite proton nuclear magnetic resonance signals.

Table S8. The number of extreme urinary metabolite concentration values in the study populations.

Supplementary figures

Figure S1. Illustration of the automated quantification models for 13 urinary metabolites.

Figure S2. Boxplots of 60 quantified urine metabolites (referenced to creatinine) in all individuals (n=994), men (n=419), and women (n=575) in the random subset of Northern Finland Birth Cohort 1966.

Figure S3. Histograms of 60 quantified urine metabolites (referenced to creatinine) in all individuals (n=994), men (n=419), and women (n=575) in the random subset of Northern Finland Birth Cohort 1966.

Figure S4. Histograms of 61 quantified urine metabolites (absolute concentrations) in all individuals (n=994), men (n=419), and women (n=575) in the random subset of Northern Finland Birth Cohort 1966.

Figure S5. Boxplots of 13 automatically quantified urinary metabolites in all the Northern Finland Birth Cohort 1966 participants (n=4505) and in the random subset (n=994).

Figure S6. Boxplots of 13 automatically quantified urinary metabolites in men and women in all the Northern Finland Birth Cohort 1966 participants (n=4505) and in the random subset (n=994).

Figure S7. Histograms of 13 automatically quantified urinary metabolites in all the Northern Finland Birth Cohort 1966 participants (n=4505) and in the random subset (n=994).

Figure S8. Histograms of 13 automatically quantified urinary metabolites in men and women in all the Northern Finland Birth Cohort 1966 participants (n=4505) and in the random subset (n=994).

Figure S9. Regression models of 12 automatically quantified urinary metabolites for BMI in Northern Finland Birth Cohort (NFBC) 1966 (n=4505), NFBC1986 (n=1010) and Cardiovascular Risk in Young Finns Study (n=474).

Figure S10. Regression models of 12 automatically quantified urinary metabolites for smoking in Northern Finland Birth Cohort (NFBC) 1966 (n=4505), NFBC1986 (n=1010) and Cardiovascular Risk in Young Finns Study (n=474).

Figure S11. Regression models of three automatically quantified urinary amino acid concentrations and their corresponding serum concentrations, estimated glomerular filtration rate (eGFR), and the multiplication of the serum concentration and eGFR in Northern Finland Birth Cohort 1966 (n=4505) and Cardiovascular Risk in Young Finns Study (n=474).

Northern Finland Birth Cohorts 1966 and 1986

The Northern Finland Birth Cohort (NFBC) studies are two longitudinal birth cohorts established to study factors affecting preterm birth and consequent morbidity in the two northernmost provinces of Finland, including Oulu and Lapland.

NFBC1966 included 12,058 children born alive into the cohort, comprising 96% of all births during 1966 in the region [1]. In 2012, at the age of 46 years, all cohort members who were alive and living in Finland with a known address (n = 10,331) were invited to participate in a large health examination that consisted of both questionnaires and clinical examination. Together they included items about social background, lifestyle (sleep, smoking, physical activity, and nutrition), medication, diagnosed diseases, organ-specific symptoms, psychiatric symptoms, workload and occupational health, economy, personal traits, functioning, quality of life, use of health services and family history of diseases. Questionnaires were received from a total of 7,146 cohort members. Basic clinical health examinations were offered by post to all cohort members and performed by three research nurse teams in 36 towns all over Finland (n=5,832). Examinations comprised, for example, anthropometric parameters, brachial blood pressure, physical performance, 15-lead electrocardiogram, heart rate variability test, pressure pain threshold and tolerance test, skin allergy test (prick test), spirometry, oral glucose tolerance test (0-, 30-, 60- and 120-min samples), cognitive test and objective measures of physical activity and sleep. Biological samples (blood, feces, urine, saliva, and hair) were obtained from all who attended clinical examination. The serum (n=5,788) and urine (n=4,549) samples were taken after overnight fasting. Participants (n=4,505) who had serum and urine metabolomics data, together with the 49 clinical and biochemical measures (1-49 in the list below), available, were included in the present study.

NFBC1986 included 9,432 children born alive between July 1st 1985 - June 30th 1986 into the cohort. The latest data collection with clinical examinations and questionnaires are at the age of 33 years. The study consisted of postal questionnaires for the whole cohort and a health examination for cohort members living in the city of Oulu and surrounding areas (250 km from Oulu) were carried out during May 2019 - December 2020. Questionnaires were received from a total of 3,587 cohort members. Basic clinical health examinations were conducted for 1,807 members. Participants

who had urine metabolomics data (n=1,010) together with 30 clinical and biochemical measures (numbers 1-11, 20, 22, 28, 30, 33, 35, 37-49 in the list below), were included in the present study. Informed written consent was obtained from all participants. The research protocols were approved by the Ethics Committee of University of Oulu and the Ethics Committee of Northern Ostrobotnia Hospital District, Finland. More information on the cohort and the data collection can be found at http://www.oulu.fi/nfbc.

Cardiovascular Risk in Young Finns Study

The Cardiovascular Risk in Young Finns Study (YFS) [2] is one of the largest follow-up studies into cardiovascular risk from childhood to adulthood. A total of 3,596 subjects participated the study in 1980. After that, several follow-up studies of this cohort have been conducted. The latest field study was conducted in 2018-2020. In addition to the original YFS participants (G1), their parents (G0) and children (G2) were invited to take part in this study phase. A total of n = 7,349 participants attended these three generational field studies. Examinations have included comprehensive data collection using questionnaires, physical measurements, and blood tests. Primary study variables have been serum lipoproteins, insulin, inflammation markers, homocysteine, obesity indices, blood pressure, life-style factors, smoking status, alcohol use, general health status, diet, physical activity, psychological factors, and socio-economic status. Participants (n=474) who had serum and urine metabolomics data, together with 39 clinical measurements (numbers 1, 6-8, 12-38, 41-42, 44-49 in the list below), were included in the present study, including 182 participants from G1, 161 from G0, and 131 from G2. All participants gave written informed consent, and the study was approved by the local ethics committees and conducted in accordance with the Declaration of Helsinki. More information on the cohort can be found at https://youngfinnsstudy.utu.fi/index.html.

Clinical and biochemical measures

Detailed descriptions of the clinical and biochemical data categories, the individual measures and the calculation of the disease risk scores are given below. * refers to clinical measurements and data, ** to quantitative serum NMR metabolomics data [3], and *** to calculated/estimated values.

Anthropometry

1) ***Body mass index**** BMI (kg/m^2) = weight (kg) / $[height (m)]^2$

2) ***Waist-to-hip ratio**** WHR = waist circumference / hip circumference

3) ***Body fat* (%) ***

4) ***Visceral fat area* ***

5) ***Total body water*** *

Blood pressure

6) ***Systolic blood pressure* ***

7) ***Diastolic blood pressure* ***

8) ***Pulse pressure* *** Pulse = systolic blood pressure – diastolic blood pressure

Fitness

9) ***Fitness score* ***

10) ***Basal metabolic rate* ***

11) ***Grip strength average* *** = (Grip strength left + Grip strength right) / 2

Amino acids

12) ***Leucine* ****

13) ***Isoleucine* ****

14) ***Valine* ****

15) ***Alanine* ****

16) ***Glutamine* ****

17) ***Glycine* ****

18) ***Phenylalanine* ****

19) ***Tyrosine* ****

Glycaemic traits & gluconeogenesis

20) ***Glycated haemoglobin (%)*** * (HbA1c)

21) ***Fasting insulin*** *

22) ***Fasting glucose*** *

23) ***Lactate*** **

24) ***Pyruvate*** **

25) ***Citrate*** **

26) ***Glycerol*** **

Lipids & lipoproteins

27) ***Apolipoprotein B*** **

28) ***Total triglycerides*** *

29) ***Apolipoprotein A-I*** **

30) ***High-density lipoprotein (HDL) cholesterol*** *

Miscellaneous blood biomarkers

31) ***Acetoacetate*** **

32) ***Beta-hydroxybutyrate*** **

33) ***C-reactive protein*** *

34) ***Glycoprotein acetyls*** **

35) ***Haemoglobin*** *

36) ***Leukocytes*** *

37) ***Platelets*** *

38) ***Erythrocytes*** *

Liver and pancreas function

39) ***Bilirubin*** *

40) ***Alkaline phosphatase*** *

41) ***Alanine aminotransferase*** *

42) ***Gamma-glutamyl transferase*** *

Kidney function

43) ***Uric acid*** *

44) ***Creatinine*** *

45) ***Estimated glomerular filtration rate (eGFR)*** *** eGFR is estimated with the Chronic Kidney Disease Epidemiology Collaboration Study equation based on serum or plasma creatinine, sex, age, and ethnicity [4] using Package 'nephro' in R.

Disease risk scores

46) ***FINRISK*** ***

The predicted outcomes for FINRISK are acute myocardial infarction and coronary heart disease mortality [5].

In men: log odds = 10.2133 – 0.0985 * age in years – 0.6072 * smoking (1 if a current smoker, 0 otherwise) – 0.0110 * systolic blood pressure in mmHg – 0.3421 * total cholesterol in mmol/l + 1.2506 * HDL-cholesterol (mmol/l) – 0.4888 * diabetes (1 if diagnosed diabetes and 0 otherwise); The 10 year risk (per cent) was calculated as 1 / (1 + exp(log odds)) * 100.

In women: log odds =10.6882 – 0.0954 * age – 0.6785 * smoking – 0.0186 * systolic blood pressure – 0.1969 * total cholesterol + 1.9039 * HDL cholesterol – 0.8799 * diabetes. The 10 year risk (per cent) was calculated as 1 / (1 + exp(log odds)) * 100.

47) ***Chronic kidney disease (CKD) Nelson*** ***

Risk prediction equations for incident CKD [6].

In nondiabetic: 1 – exp (-5^1.055408 × exp [-3.609661 + 0.2582196 × (age/5 – 11) + 0.1821665 × (if female) + 0.1808945 × (if black) + 0.4581006 × (15 – min(eGFR, 90)/5) – 0.3159218 × max(0, eGFR-90)/5 + 0.1953927 × (if has history of cardiovascular disease (CVD)) + 0.1213741 × (if ever smoking) + 0.3543645 × (if hypertensive) + 0.0630538 × (BMI/5-5.4) + 0.3519087 × (log10 (albumin-creatinine ratio (ACR)) - expected log10ACR*)])

In diabetic: 1 – exp (-5^0.9766551 × exp [-2.647004 + 0.1351572 × (age/5 – 11) + 0.1381975 × (if female) + 0.0920208 × (if black) + 0.3546697 × (15 – min(eGFR, 90)/5) – 0.1525133 × max(0, eGFR-90)/5 + 0.1870637 × (if has history of CVD) + 0.0619679 × (HbA1c –7) + 0.1078296 × (if insulin use) – 0.150944 × (if no diabetes

mellitus (DM) medication use) + 0.023959 × (HbA1c –7) × (if insulin use) +0.0398424 × (HbA1c –7) × (if no DM medication use) – 0.00084 × (if ever smoking) + 0.3653268 × (if hypertensive) + 0.050306 × (BMI/5-5.4) + 0.3737905 × (log10ACR – 1)])

If insulin in use: For diabetes are positive (only 22 type 2 diabetes cases and 2 type 1 diabetes cases), others are negative.

If no DM medication in use: For diabetes are negative and normal people are positive.

If hypertensive: Hypertension was defined as blood pressure more than 140/90 mm Hg.

Due to the good health and youth in most participants, some approximations were used in these calculations: History of CVD: no cases; ACR: 0 in all individuals.

48) ***CKD O'Seaghdha*** ***

A risk score for CKD in the general population [7].

1 – (1 – 0.092) ^ 0.5 / ((1 – 0.092) ^ 0.5 + (1 – (1 – 0.092) ^ 0.5) × exp (- 6.235 + 0.095 × age + 0.476 × (if diabetics) + 0.761 × (if hypertensive) + 0.779 × (if 75 ≤ eGFR < 90) + 1.558 × (if 60 ≤ eGFR <75) + 0.300 × (if ACR ≥ 30 or dipstick ≥ trace)))

Approximation: ACR: 0 in all individuals.

49) ***CKD Chien*** ***

A prediction model for the risk of incident CKD [8].

1 – 0.9632 ^ exp (-6.8 + 0.077 × age + 0.366 × (if diabetics) + 1.24 × (if history of stroke) + 0.059 × BMI + 0.018 × systolic blood pressure)

Approximation: History of stroke: no cases.

Supplementary tables

Table S1. Sex specific characteristics of the Northern Finland Birth Cohort 1966.

**Characteristics**	**Men**	**Women**	***P***
Number of participants	1950	2555	-
Age (years)	46.7 (46.3-47.1)	46.7 (46.2-47.1)	0.2
Body mass index (kg/m2)	27 (24-29)	25 (23-29)	3.0E-22
Waist-to-hip ratio	0.98 (0.94-1.02)	0.86 (0.82-0.9)	p<5.0E-324
Body fat (%)	23 (18-28)	33 (27-39)	3.3E-288
Visceral fat area (cm^2)	95 (73-121)	102 (79-131)	9.0E-13
Total body water (L)	48 (45-52)	34 (32-37)	p<5.0E-324
Systolic blood pressure (mmHg)	129 (121-139)	119 (110-130)	5.8E-104
Diastolic blood pressure (mmHg)	86 (79-94)	82 (75-89)	2.2E-38
Pulse (bpm)	67 (60-75)	71 (64-78)	1.4E-27
Fitness score	77 (72-82)	72 (67-77)	9.5E-115
Basal metabolic rate (kilocalories)	1785 (1681-1901)	1376 (1301-1455)	p<5.0E-324
Grip strength average (kg)	46 (41-52)	27 (23-30)	p<5.0E-324
Smoking prevalence (%)	20	15.8	-
Leucine (mmol/L)	0.10 (0.09-0.11)	0.08 (0.07-0.08)	p<5.0E-324
Isoleucine (mmol/L)	0.06 (0.05-0.08)	0.05 (0.04-0.06)	3.0E-291
Valine (mmol/L)	0.22 (0.20-0.25)	0.18 (0.16-0.21)	4.7E-263
Alanine (mmol/L)	0.46 (0.42-0.51)	0.45 (0.41-0.5)	8.0E-10
Glutamine (mmol/L)	0.59 (0.55-0.64)	0.55 (0.51-0.59)	1.4E-110
Glycine (mmol/L)	0.27 (0.25-0.3)	0.30 (0.27-0.36)	5.5E-89
Phenylalanine (mmol/L)	0.08 (0.07-0.09)	0.08 (0.07-0.08)	2.2E-45
Tyrosine (mmol/L)	0.06 (0.05-0.07)	0.05 (0.05-0.06)	2.9E-91
Glycated haemoglobin (%)	5.5 (5.3-5.8)	5.4 (5.2-5.7)	2.7E-22
Fasting insulin (IU/L)	8.7 (5.8-12.8)	7.4 (5.2-10.7)	7.6E-17
Fasting glucose (mmol/L)	5.6 (5.3-5.9)	5.3 (5.0-5.6)	2.6E-109
Lactate (mmol/L)	1.5 (1.3-1.8)	1.4 (1.2-1.6)	1.3E-42
Pyruvate (mmol/L)	0.10 (0.08-0.12)	0.09 (0.08-0.12)	0.89
Citrate (mmol/L)	0.12 (0.11-0.13)	0.12 (0.11-0.13)	0.0040

Characteristics	Men	Women	*P*
Glycerol (mmol/L)	0.07 (0.06-0.09)	0.08 (0.06-0.10)	1.6E-05
Apolipoprotein B (g/L)	1.13 (0.97-1.3)	0.95 (0.83-1.11)	2.7E-117
Total triglycerides (mmol/L)	1.23 (0.89-1.73)	0.92 (0.71-1.24)	3.0E-81
Apolipoprotein A-I (g/L)	1.7 (1.5-1.8)	1.8 (1.7-1.9)	2.8E-81
HDL cholesterol (mmol/L)	1.3 (1.2-1.6)	1.6 (1.4-1.9)	2.4E-131
Acetoacetate (mmol/L)	0.04 (0.03-0.06)	0.03 (0.03-0.05)	3.4E-15
Beta-hydroxybutyrate (mmol/L)	0.13 (0.11-0.16)	0.12 (0.10-0.16)	1.4E-10
C-reactive protein (mg/L)	0.79 (0.46-1.5)	0.84 (0.45-1.8)	0.033
Glycoprotein acetyls (mmol/L)	1.4 (1.3-1.6)	1.4 (1.2-1.5)	5.4E-28
Haemoglobin (g/L)	151 (145-157)	134 (127-140)	p<5.0E-324
Leukocytes (× 10*9 cells/L)	5.3 (4.5-6.3)	5.4 (4.5-6.5)	0.11
Platelets (× 10*9 cells/L)	231 (204-261)	264 (228-303)	3.8E-89
Erythrocytes (× 10*12 cells/L)	4.9 (4.7-5.2)	4.5 (4.2-4.7)	p<5.0E-324
Bilirubin (μmol/L)	12 (10-16)	11 (8-14)	3.0E-28
Alkaline phosphatase (U/L)	66 (56-78)	58 (48-70)	1.8E-56
Alanine aminotransferase (U/L)	34 (26-46)	20 (16-27)	8.0E-255
Gamma-glutamyl transferase (U/L)	33 (22-52)	17 (13-27)	7.1E-200
Uric acid (μmol/L)	349 (308-397)	261 (226-300)	p<5.0E-324
Creatinine (μmol/L)	75 (69-83)	62 (56-67)	p<5.0E-324
eGFR (mL/min/1.73m^2)	104 (97-108)	104 (94-107)	1.9E-11
CVD FINRISK risk	1.83 (1.21-2.69)	0.23 (0.13-0.4)	p<5.0E-324
CKD Nelson risk	1.3 (0.9-2.0)	1.5 (1.1-2.7)	8.1E-28
CKD O'Seaghdha risk	1.22 (0.76-1.61)	0.76 (0.76-1.61)	5.0E-05
CKD Chien risk	7.0 (5.5-9.3)	5.5 (4.2-7.6)	3.6E-70

Values are median (interquartile range).
Smoking prevalence = number of current smokers / number of cohort participants.
Wilcoxon's Signed Rank Test for the comparisons between men and women.
Abbreviations: HDL, high-density lipoprotein; eGFR, estimated glomerular filtration rate; CVD, Cardiovascular disease CKD, chronic kidney disease; FINRISK, a large Finnish population survey on risk factors on chronic, noncommunicable diseases.

Table S2. Sex specific characteristics of the Northern Finland Birth Cohort 1986.

Characteristics	Men	Women	*P*
Number of participants	427	583	-
Age (years)	33.8 (33.4-34.1)	33.7 (33.4-34.1)	0.58
Body mass index (kg/m^2)	25 (24-28)	24 (22-28)	0.00015
Waist-to-hip ratio	0.91 (0.87-0.97)	0.92 (0.87-0.97)	0.87
Body fat (%)	20 (16-25)	31 (25-38)	4.2E-65
Visceral fat area (cm^2)	73 (53-99)	93 (64-143)	6.5E-13
Total body water (L)	48 (44-51)	34 (31-37)	3.3E-144
Systolic blood pressure (mmHg)	119 (112-127)	105 (99-113)	7.4E-65
Diastolic blood pressure (mmHg)	75 (71-81)	72 (66-78)	1.6E-11
Pulse (bpm)	69 (61-76)	74 (67-81)	1.1E-13
Fitness score	79 (74-84)	73 (68-78)	2.0E-32
Basal metabolic rate (kilocalories)	1776 (1679-1880)	1370 (1295-1469)	5.1E-144
Grip strength average (kg)	48 (43-53)	30 (27-33)	3.3E-148
Smoking prevalence (%)	16.4	12.4	-
Glycated haemoglobin (%)	5.2 (5.0-5.4)	5.2 (5.0-5.4)	0.032
Fasting glucose (mmol/L)	5.2 (4.9-5.4)	4.8 (4.6-5.1)	5.6E-38
Total triglycerides (mmol/L)	0.89 (0.66-1.2)	0.72 (0.54-0.99)	1.8E-10
HDL cholesterol (mmol/L)	1.3 (1.1-1.5)	1.5 (1.3-1.8)	2.5E-25
C-reactive protein (mg/L)	0.58 (0.30-1.20)	0.88 (0.40-1.94)	5.2E-07
Haemoglobin (g/L)	147 (142-153)	130 (124-135)	3.2E-125
Platelets (× 10*9 cells/L)	230 (203-261)	248 (216-287)	9.4E-10
Erythrocytes (× 10*12 cells/L)	4.9 (4.7-5.1)	4.3 (4.2-4.6)	3.5E-105
Bilirubin (μmol/L)	14 (11-18)	11 (9-14)	1.1E-13

Characteristics	Men	Women	*P*
Alkaline phosphatase (U/L)	60 (51-71)	51 (43-62)	7.0E-17
Alanine aminotransferase (U/L)	30 (22-43)	17 (14-22)	6.1E-66
Gamma-glutamyl transferase (U/L)	21 (15-31)	13 (10-16)	4.2E-47
Uric acid (μmol/L)	355 (309-395)	264 (228-310)	1.2E-72
Creatinine (μmol/L)	74 (68-81)	60 (54-65)	4.8E-93
eGFR (mL/min/1.73m^2)	115 (109-118)	115 (106-118)	0.43
CVD FINRISK risk	0.38 (0.27-0.52)	0.05 (0.03-0.08)	9.4E-125
CKD Nelson risk	0.26 (0.21-0.42)	0.31 (0.25-0.53)	5.7E-08
CKD O'Seaghdha risk	0.24 (0.23-0.26)	0.24 (0.23-0.25)	0.061
CKD Chien risk	2.0 (1.7-2.6)	1.5 (1.2-2.0)	9.6E-34

Values are median (interquartile range).

Smoking prevalence = number of current smokers / number of cohort participants.

Wilcoxon's Signed Rank Test for the comparisons between men and women.

Abbreviations: HDL, high-density lipoprotein; eGFR, estimated glomerular filtration rate; CVD, Cardiovascular disease CKD, chronic kidney disease; FINRISK, a large Finnish population survey on risk factors on chronic, noncommunicable diseases.

Table S3. Sex specific characteristics of the Cardiovascular Risk in Young Finns Study.

**Characteristics**	**Men**	**Women**	***P***
Number of participants	181	293	-
Age (years)	50.5 (41-70)	49.9 (29.8-66.7)	0.17
Body mass index (kg/m^2)	27 (25-30)	27 (24-30)	0.80
Systolic blood pressure (mmHg)	133 (123-145)	125 (115-139)	1.3E-05
Diastolic blood pressure (mmHg)	79 (73-86)	77 (70-83)	0.0063
Pulse (bpm)	66 (59-74)	70 (65-77)	5.5E-05
Smoking prevalence (%)	19.1	18.4	-
Leucine (mmol/L)	0.14 (0.12-0.15)	0.11 (0.10-0.12)	3.6E-25
Isoleucine (mmol/L)	0.07 (0.05-0.07)	0.05 (0.05-0.06)	1.3E-20
Valine (mmol/L)	0.25 (0.23-0.28)	0.22 (0.19-0.24)	2.7E-17
Alanine (mmol/L)	0.38 (0.33-0.44)	0.37 (0.32-0.42)	0.12
Glutamine (mmol/L)	0.78 (0.74-0.83)	0.73 (0.68-0.78)	8.2E-13
Glycine (mmol/L)	0.25 (0.22-0.29)	0.27 (0.23-0.33)	0.0031
Phenylalanine (mmol/L)	0.06 (0.05-0.07)	0.06 (0.05-0.06)	0.021
Tyrosine (mmol/L)	0.07 (0.06-0.07)	0.06 (0.05-0.07)	0.00045
Glycated haemoglobin (%)	5.5 (5.3-5.8)	5.5 (5.3-5.8)	0.47
Fasting insulin (IU/L)	9.2 (4.6-13.7)	9.1 (5.6-13.1)	0.31
Fasting glucose (mmol/L)	5.4 (5.2-5.9)	5.3 (5.0-5.6)	6.3E-05
Lactate (mmol/L)	2.2 (1.8-2.5)	2.0 (1.7-2.4)	0.015
Pyruvate (mmol/L)	0.06 (0.05-0.08)	0.07 (0.05-0.08)	0.017
Citrate (mmol/L)	0.04 (0.04-0.05)	0.04 (0.04-0.05)	0.84
Glycerol (mmol/L)	0.10 (0.07-0.12)	0.13 (0.10-0.16)	5.7E-20
Apolipoprotein B (g/L)	0.92 (0.77-1.07)	0.94 (0.79-1.12)	0.32
Total triglycerides (mmol/L)	1.14 (0.89-1.61)	1.11 (0.79-1.5)	0.042
Apolipoprotein A-I (g/L)	1.4 (1.3-1.5)	1.6 (1.4-1.8)	1.9E-15
HDL cholesterol (mmol/L)	1.2 (1-1.4)	1.4 (1.2-1.7)	5.7E-14

**Characteristics**	**Men**	**Women**	***P***
Acetoacetate (mmol/L)	0.02 (0.01-0.04)	0.03 (0.01-0.04)	0.61
Beta-hydroxybutyrate (mmol/L)	0.05 (0.02-0.11)	0.05 (0.02-0.11)	0.98
C-reactive protein (mg/L)	1.01 (0.50-1.9)	1.04 (0.55-2.48)	0.24
Glycoprotein acetyls (mmol/L)	0.89 (0.81-0.95)	0.89 (0.81-0.96)	0.80
Haemoglobin (g/L)	154 (147-162)	140 (133-145)	2.5E-38
Leukocytes (× 10*9 cells/L)	6.0 (5.2-7.3)	6.1 (5.2-7.1)	0.81
Platelets (× 10*9 cells/L)	237 (207-269)	265 (225-305)	6.0E-07
Erythrocytes (× 10*12 cells/L)	5 (4.8-5.3)	4.6 (4.4-4.8)	1.7E-25
Alanine aminotransferase (U/L)	28 (21-38)	19 (14-25)	1.6E-14
Gamma-glutamyl transferase (U/L)	31 (23-46)	20 (16-29)	1.1E-15
Creatinine (μmol/L)	88 (79-96)	70 (65-77)	1.5E-35
eGFR (mL/min/1.73m^2)	88 (75-99)	86 (74-101)	0.89
CVD FINRISK risk	4.22 (0.91-13.97)	0.50 (0.09-3.18)	3.2E-17
CKD Nelson risk	6.6 (1.5-28.6)	6.6 (0.9-34.3)	0.72
CKD O'Seaghdha risk	4.18 (1.02-26.85)	3.02 (0.31-17.52)	0.037
CKD Chien risk	11.5 (3.6-43.7)	9.3 (1.9-36.6)	0.045

Values are median (interquartile range).

Smoking prevalence = number of current smokers / number of cohort participants.

Wilcoxon's Signed Rank Test for the comparisons between men and women.

Abbreviations: HDL, high-density lipoprotein; eGFR, estimated glomerular filtration rate; CVD, Cardiovascular disease CKD, chronic kidney disease; FINRISK, a large Finnish population survey on risk factors on chronic, noncommunicable diseases.

Table S4. Intra-assay variation as well as intra-individual and inter-individual variation of quantified urine metabolites.

Metabolite	Intra-assay CV (%)[a,b]	Intra-individual CV (%)[a,c]	Inter-individual CV (%)[a,d]
**Amino acids**			
Alanine	1.16	28.69	49.85
Glycine	2.21	34.71	73.99
Histidine	1.10	30.25	48.75
Threonine	4.57	38.58	75.55
Isoleucine	6.68	23.27	53.56
Leucine	6.34	26.48	58.25
Valine	4.72	20.28	39.22
Tryptophan	3.34	33.76	49.66
Tyrosine	3.35	32.09	45.76
**Metabolism of amino acids**			
Betaine	3.23	43.96	176.13
Creatine	4.12	126.19	240.36
3-Hydroxyisobutyrate	2.67	34.18	61.42
Hippurate	1.15	58.45	69.29
Phenylacetylglutamine	3.12	31.90	54.00
Urea	1.46	32.98	39.41
Pyroglutamate	4.40	17.64	27.64
**Carbohydrate metabolism**			
Glucose	2.91	13.76	1294.34
Lactate	4.28	44.26	477.64
Citrate	1.51	27.92	53.37
Cis-aconitate	0.85	22.28	39.64
Fumarate	-[e]	176.23	218.72
Succinate	13.36	32.08	179.91
Mannitol	- [e]	164.85	222.25
Glucuronate	4.07	18.31	66.41
Hypoxanthine	3.53	38.80	73.70
Pseudouridine	2.15	6.32	14.28
Uracil	4.29	37.71	148.66
**Nicotinate and NAM**			
2-PY	2.14	35.29	60.78
N1-Methylnicotinamide	1.32	28.24	52.30
Trigonelline	0.79	68.71	74.78
**Caffeine metabolism**			
3-Methylxanthine	5.02	174.27	84.08
**Microbial metabolism**			
3-Hydroxyhippurate	2.56	51.81	100.10

4-Hydroxyhippurate	3.43	34.85	71.88
4-Hydroxyphenylacetate	2.24	28.73	52.08
Acetate	14.17	62.87	394.40
Dimethylamine	0.74	9.79	30.50
Formate	8.71	41.32	587.34
HPHPA	4.30	67.68	99.97
Methanol	1.91	60.33	114.21
Methylamine	3.17	32.07	49.79
p-Cresol sulphate	1.53	35.65	71.32
Trimethylamine *N*-oxide	1.63	80.89	127.18
**Modification of histones**			
2-Hydroxyisobutyrate	1.15	16.25	35.39
**Dietary metabolites**			
1-Methylhistidine	2.04	21.15	31.26
2-Furoylglycine	5.46	225.45	212.89
3-Methylhistidine	1.56	95.44	117.02
Arabinose	3.58	35.50	59.28
Indoxyl sulphate	1.59	32.24	46.79
Levoglucosan	1.52	304.04	190.10
Proline-betaine	2.71	132.03	139.93
Quinate	3.51	262.56	81.67
Scyllitol	1.19	22.05	57.91
Sucrose	4.45	194.15	555.87
Trans-ferulate	4.71	31.12	101.66
Xylose	3.38	99.60	112.05
**Miscellaneous**			
3-Hydroxyisovalerate	4.84	66.55	46.13
4-Deoxyerythronate	1.59	18.15	38.46
4-Deoxythreonate	1.67	30.42	38.60
Sumiki's acid	2.36	35.23	133.83
Trans-aconitate	4.42	50.71	59.02

[a]Concentrations are scaled to the concentration of creatinine; coefficients of variation percent = (standard deviation / average) * 100%

[b]One urine sample prepared and analysed as 10 replicates; reflects the entire quantitative process, i.e., including all the sample preparation steps, nuclear magnetic resonance experimentation and mathematical quantification protocols.

[c]A 30-day consecutive urine collection, averaged over 3 different volunteers.

[d]One thousand and three different individuals from the Northern Finland Birth Cohort 1966.

[e]Concentration of the metabolite below the detection limit in this urine sample.

Abbreviations: 2-PY, N1-Methyl-2-pyridone-5-carboxamide; HPHPA, 3-(3-Hydroxyphenyl)-3-hydroxypropanoate; NAM, nicotinamide; CV, coefficients of variation.

Table S5. Concentrations for 61 urinary metabolites in men and women in the random subset of Northern Finland Birth Cohort 1966 (n=994).

	**Absolute concentrations (μmol/L)**			**Creatinine-referenced concentrations (μM/mM creatinine)**		
**Metabolites**	**Men (n=419)**	**Women (n=575)**	***P***	**Men (n=419)**	**Women (n=575)**	***P***
**Amino acids**						
Alanine	246 [172-369]	191 [131-311]	7.7E-08	18.90 [14.19-26.47]	20.78 [15.96-28.68]	1.9E-03
Glycine	932 [647-1437]	1101 [724-1861]	2.1E-05	73.60 [50.74-103.6]	124 [82.6-185]	1.0E-38
Histidine	976 [667-1421]	652 [406-1067]	2.4E-21	76.43 [56.42-107.6]	68.98 [47.15-99.54]	5.7E-04
Threonine	107 [68-155]	89 [57-139]	2.8E-04	8.29 [5.61-12.26]	9.83 [6.73-13.15]	1.5E-05
Isoleucine	10 [7-15]	9 [7-14]	1.8E-03	0.82 [0.65-1.08]	0.96 [0.74-1.24]	1.4E-05
Leucine	26 [19-36]	20 [14-30]	3.9E-13	2.09 [1.64-2.64]	2.09 [1.65-2.63]	5.0E-01
Valine	48 [26-50]	30 [21-43]	3.5E-10	2.92 [2.34-3.65]	3.10 [2.55-3.88]	1.4E-03
Tryptophan	76 [51-115]	61 [39-95]	3.6E-10	6.09 [4.64-8.19]	6.23 [4.47-8.56]	9.1E-01
Tyrosine	138 [97-196]	92 [60-144]	2.1E-22	10.91 [8.21-14.38]	9.87 [7.21-13.21]	4.8E-04
**Metabolism of amino acids**						
Betaine	69 [41-119]	58 [37-96]	5.4E-03	5.18 [3.36-8.62]	6.47 [4.08-10.32]	3.6E-04
Creatine	116 [70-223]	158 [77-591]	8.9E-06	8.21 [6.14-14.08]	14.52 [7.90-55.39]	9.8E-19
3-Hydroxyisobutyrate	128 [80-187]	108 [70-164]	3.2E-04	9.33 [7.10-12.63]	10.87 [8.20-14.70]	1.9E-06
Hippurate	4774 [2724-7843]	4688 [2600-7881]	9.5E-01	380 [229-613]	502 [289-762]	5.7E-09
Urea	296129 [246165-346324]	257252 [212231-308767]	1.1E-12	23110 [18371-29362]	27650 [20610-34328]	1.2E-09
Pyroglutamate	263 [202-330]	238 [181-302]	6.1E-06	20.69 [17.56-23.84]	24.55 [20.36-28.99]	3.5E-24
**Carbohydrate metabolism**						
Glucose	295 [217-390]	241 [178-343]	1.1E-08	22.62 [19.54-26.42]	25.33 [21.66-30.41]	8.9E-11
Lactate	76 [48-116]	74 [46-123]	8.4E-01	5.52 [3.94-7.79]	7.40 [5.38-11.14]	1.0E-16
Citrate	1716 [1090-2612]	2366 [1506-3470]	4.4E-12	140 [91-195]	251 [177-332]	2.9E-56

Cis-aconitate	221 [154-308]	190 [134-273]	5.8E-04	17.27 [13.64-20.90]	19.77 [16.34-24.41]	1.0E-13
Fumarate	6 [4-10]	7 [4-11]	2.0E-02	0.40 [0.27-0.65]	0.65 [0.45-0.99]	2.6E-22
Succinate	28 [19-44]	42 [25-68]	2.5E-16	2.21 [1.57-3.24]	4.45 [3.05-6.28]	4.7E-58
Mannitol	448 [244-917]	437 [233-878]	6.3E-01	34.32[19.07-69.43]	49.27 [25.83-91.47]	4.9E-01
**Nucleotide metabolism**						
Glucuronate	277 [200-356]	244 [178-342]	2.1E-03	20.64 [17.55-24.93]	24.57 [20.89-30.04]	3.4E-20
Hypoxanthine	41 [25-67]	33 [19-54]	1.7E-06	3.15 [2.24-4.76]	3.28 [2.29-4.96]	2.2E-01
Pseudouridine	343 [264-441]	295 [212-414]	1.3E-06	26.55 [24.89-28.28]	30.65 [28.21-32.92]	1.6E-63
Uracil	58 [39-84]	60 [41-92]	1.9E-01	4.59 [3.28-6.77]	6.47 [4.75-8.74]	7.9E-18
**Nicotinate and nicotinamide metabolism**						
2-PY	167 [110-247]	133 [83-203]	4.8E-08	12.47 [9.53-17.76]	13.48 [9.03-19.02]	2.3E-01
N1-Methylnicotinamide	56 [37-85]	47 [31-69]	1.8E-06	4.37 [3.12-6.35]	4.78 [3.42-6.73]	1.0E-02
Trigonelline	921 [463-1508]	802 [396-1373]	3.9E-02	70.70 [38.71-120.8]	86.44 [46.20-139]	2.7E-03
**Caffeine metabolism**						
3-Methylxanthine	91 [59-140]	94 [53-156]	6.8E-01	7.10 [4.93-10.19]	9.71 [6.18-14.58]	1.1E-12
**Microbial metabolism**						
3-Hydroxyhippurate	592 [272-1112]	449 [192-875]	4.5E-05	51.26 [23.75-90.10]	47.54 [21.45-89.35]	9.3E-01
4-Hydroxyhippurate	94 [64-144]	96 [59-157]	6.3E-01	7.11 [5.10-10.63]	10.22 [6.92-14.52]	1.5E-17
4-Hydroxyphenylacetate	179 [126-249]	151 [99-219]	9.1E-07	13.26 [10.40-18.38]	14.85 [11.95-19.94]	1.3E-04
Acetate	48 [34-72]	47 [32-74]	7.8E-01	3.78 [2.67-5.60]	4.86 [3.22-7.91]	1.1E-10
Dimethylamine	402 [297-507]	329 [233-457]	3.3E-09	29.61 [27.31-32.24]	32.91 [30.60-36.45]	3.2E-34
Formate	265 [179-360]	216 [148-314]	3.1E-06	19.92 [14.54-30.23]	22.92 [15.88-32.73]	1.3E-03
HPHPA	327 [158-564]	323 [160-575]	6.9E-01	27.31 [14.32-45.34]	33.97 [17.44-58.68]	3.1E-02
Indoxyl sulfate	332 [227-476]	330 [210-510]	8.2E-01	25.67 [19.85-33.91]	33.90 [25.25-45.12]	6.9E-20
Methanol	31 [23-43]	30 [23-42]	5.0E-01	2.44 [1.72-3.66]	3.21 [2.05-5.25]	2.4E-09
Methylamine	32 [20-48]	28 [17-45]	8.3E-03	2.45 [1.80-3.20]	2.78 [1.98-3.80]	2.7E-05
p-Cresol sulfate	299 [165-483]	372 [205-619]	6.3E-05	24.08 [13.70-37.70]	38.45 [23.23-59.16]	2.0E-21

Phenylacetylglutamine	717 [452-1030]	706 [442-1150]	8.4E-01	56.50 [38.35-76.29]	75.40 [52.53-101]	1.1E-16
Trimethylamine N-oxide	515 [329-746]	418 [265-650]	3.0E-05	36.82 [27.37-54.29]	39.98 [29.54-64.38]	6.3E-03
**Modification of histones**						
2-Hydroxyisobutyrate	55 [40-78]	45 [30-65]	6.4E-11	4.30 [3.59-5.20]	4.59 [3.66-5.68]	8.8E-03
**Dietary metabolites**						
1-Methylhistidine	383 [287-496]	277 [203-369]	1.3E-25	29.08 [24.49-34.48]	27.94 [22.63-34.06]	2.7E-03
2-Furoylglycine	57 [32-112]	48 [27-83]	2.6E-04	4.60 [2.75-7.96]	4.55 [2.69-8.35]	1.5E-02
3-Methylhistidine	506 [214-1180]	416 [179-1036]	1.8E-02	38.05 [18.40-87.78]	45.40 [18.58-102]	3.1E-01
Arabinose	117 [82-172]	103 [70-159]	2.5E-03	9.05 [6.99-11.68]	10.95 [8.53-14.15]	1.3E-12
Levoglucosan	94 [61-162]	86 [57-170]	5.0E-01	7.41 [6.64-12.15]	9.20 [5.66-16.53]	9.2E-01
Proline-betaine	200 [70-455]	173 [51-493]	3.1E-01	15.76 [5.68-36.69]	17.94 [5.21-51.71]	2.1E-02
Quinate	301 [208-437]	283 [191-428]	7.1E-02	24.25 [18.04-34.58]	30.37 [21.21-41.10]	5.3E-04
Scyllitol	66 [44-99]	69 [45-108]	2.8E-01	5.06 [3.73-7.16]	7.06 [5.09-9.87]	4.7E-23
Sucrose	40 [21-87]	39 [21-88]	9.5E-01	3.20 [1.64-6.36]	4.17 [2.27-8.22]	5.0E-05
Trans-ferulate	24 [18-32]	23 [16-32]	3.5E-01	1.69 [1.35-2.11]	2.09 [1.59-2.78]	3.2E-05
Xylose	71 [49-117]	71 [46-118]	6.8E-01	5.33 [3.77-8.71]	6.99 [5.06-11.94]	1.8E-11
**Miscellaneous**						
3-Hydroxyisovalerate	70 [48-98]	57 [38-84]	4.0E-07	5.33 [4.24-6.91]	5.81 [4.65-7.45]	3.6E-04
4-Deoxyerythronate	125 [86-163]	95 [64-138]	3.7E-10	9.15 [7.36-11.68]	9.94 [7.54-12.71]	4.3E-03
4-Deoxythreonate	281 [195-422]	188 [131-263]	2.8E-30	21.62 [17.67-28.62]	18.53 [15.20-23.70]	6.3E-12
Sumiki's acid	38 [18-74]	29 [15-59]	9.6E-04	2.94 [1.45-5.69]	2.94 [1.64-5.69]	3.9E-01
Trans-aconitate	57 [38-79]	45 [31-68]	2.2E-07	4.43 [3.57-5.31]	4.57 [3.75-5.91]	4.9E-03
Creatinine	13109 [10042-16367]	9567 [6927-13530]	2.9E-20	1000	1000	/

Values are median [interquartile range].

Wilcoxon’s Signed Rank Test for the comparisons between men and women.

Abbreviations: 2-PY, N1-Methyl-2-pyridone-5-carboxamide; HPHPA, 3-(3-Hydroxyphenyl)-3-hydroxypropanoate.

Table S6. Concentrations for 13 urinary metabolites in Northern Finland Birth Cohort (NFBC) 1966, NFBC1986 and Cardiovascular Risk in Young Finns Study.

**Metabolites (µM/mM creatinine)**	**NFBC1966 (n=4505)**	**NFBC1986 (n=1010)**	**YFS (n=474)**
2-Hydroxyisobutyrate	4.5 [3.64-5.52]	4.27 [3.51-5.39]	5.75 [4.59-7.23]
Valine	3.09 [2.49-3.86]	2.82 [2.28-3.55]	3.04 [2.44-3.86]
Alanine	20.71 [15.42-28.28]	17.61 [13.68-24.52]	24.84 [18.93-35.15]
Pseudouridine	29.44 [26.16-33.46]	28.52 [25.06-32.92]	31.73 [27.85-36.01]
Glucose	24.17 [20.6-28.83]	22.38 [19-26.58]	29.37 [23.91-41.52]
Dimethylamine	31.85 [29.02-35.39]	31.93 [28.88-35.69]	34.2 [30.52-39.36]
Glycine	97.85 [66.16-152.45]	90.77 [60.81-140.47]	99.63 [66.78-150.9]
Citrate	201.62 [134.64-289.16]	153.78 [95.85-232.79]	263.72 [175.51-390.45]
Urea	26118 [19805-33823]	25356 [19130-33108]	29186 [21519-39244]
Formate	22.39 [15.58-32.06]	21.54 [15.46-30.22]	23.45 [16.34-34.51]
Trigonelline	82.89 [42.59-133.82]	58.48 [25.26-101.88]	47.28 [15.95-102.71]
Hippurate	466.84 [267.05-705.55]	364.13 [212.24-579.65]	323.41 [175.92-559.13]
Creatinine (mmol/L)	10.79 [7.5-14.83]	9.24 [6.37-13.28]	6.47 [2.98-11.9]

Values are median [interquartile range].

The concentrations are referenced to urinary creatinine, except for creatinine.

Abbreviations: NFBC, Northern Finland Birth Cohort; YFS, Cardiovascular Risk in Young Finns Study.

Table S7. Correction coefficients and chemical shifts of the quantified urine metabolite proton nuclear magnetic resonance signals.[a]

Metabolite	Correction coefficient	Chemical shift[b] of the quantified signal (ppm)
Creatinine	0.89	4.06
**Amino acids**		
Alanine	0.90	1.49
Glycine	0.95	3.57
Histidine	1.00	7.95-8.32
Threonine	0.83	1.34
Isoleucine	0.88	1.02
Leucine	0.88	0.96
Valine	0.88	1.05
Tryptophan	0.91	7.72
Tyrosine	0.94	6.90
**Metabolism of amino acids**		
Betaine	0.89	3.27
Creatine	0.89	3.03
3-Hydroxyisobutyrate	0.86	1.08
Hippurate	1.05	7.64
Phenylacetylglutamine	0.83	2.28
Urea	20.15[c]	5.81
Pyroglutamate	0.92	2.41
**Carbohydrate metabolism**		
Glucose	0.90	4.67
Lactate	0.86	1.34
Citrate	0.89	2.70
Cis-aconitate	1.02	5.78
Fumarate	1.16	6.53
Succinate	0.92	2.42
Mannitol	0.83	3.88
Glucuronate	0.90	4.66
Hypoxanthine	1.05	8.17
Pseudouridine	0.87	7.68
Uracil	1.26	5.80
**Nicotinate and NAM**		
2-PY	1.13	6.67
N1-Methylnicotinamide	0.97	4.47
Trigonelline	0.97	4.43
**Caffeine metabolism**		
3-Methylxanthine	1.04	8.03

**Microbial metabolism**		
3-Hydroxyhippurate	1.04	7.12
4-Hydroxyhippurate	0.95	7.76
4-Hydroxyphenylacetate	1.04	6.87
Acetate	1.19	1.93
Dimethylamine	1.06	2.73
Formate	1.60	8.46
HPHPA	0.88	5.03
Methanol	1.33	3.37
Methylamine	1.08	2.61
p-Cresol sulfate	0.96	2.34
Trimethylamine *N*-oxide	0.90	3.28
**Modification of histones**		
2-Hydroxyisobutyrate	0.89	1.37
**Dietary metabolites**		
1-Methylhistidine	1.04	7.05-7.16
2-Furoylglycine	1.13	6.65
3-Methylhistidine	0.89	8.06-8.48
Arabinose	0.90	4.54
Indoxyl sulfate	1.20	7.70
Levoglucosan	1.15	5.47
Proline-betaine	0.91	3.11
Quinate	0.85	1.88
Scyllitol	0.96	3.36
Sucrose	0.91	5.42
Trans-ferulate	0.88	6.41
Xylose	0.87	4.59
**Miscellaneous**		
3-Hydroxyisovalerate	0.87	1.28
4-Deoxyerythronate	0.88	1.11
4-Deoxythreonate	0.89	1.24
Sumiki's acid	0.86	4.61
Trans-aconitate	1.03	6.61

[a]The relaxation time used in the measurements is rather short, affecting the areas of the metabolites signals, as well as the concentration reference TSP (3-(trimethylsilyl)propionic-2,2,3,3 acid D4), in different ways depending on the relaxation time of the protons in question. Thus, we determined the correction coefficients for each quantified metabolite (**Table S7**) to lead to the true absolute metabolite concentrations. The correction coefficients were obtained by measuring a set of Northern Finland Birth Cohort 1966 urine samples also with a long relaxation delay and comparing the resulting concentrations to those obtained via the high-throughput parameters.

[b]Most of the metabolite chemical shifts depend on ion concentrations and/or sample pH and vary between urine samples. Thus, and average chemical shift, or in the case of extremely mobile signals, a chemical shift range, is given.

[c]The correction coefficient of urea considers both the effect of the relatively short relaxation delay during the nuclear magnetic resonance measurement and the fact that the urea signal is arising from NH protons that are in rapid exchange with solvent hydrogen and deuterium atoms, and thus, the observed urea signal area in the proton nuclear magnetic resonance spectrum underestimates the real urea concentration.

Abbreviations: NAM, nicotinamide; 2-PY, N1-Methyl-2-pyridone-5-carboxamide; HPHPA, 3-(3-Hydroxyphenyl)-3-hydroxypropanoate.

Table S8. The number of extreme urinary metabolite concentration values in the study populations.

	Number of extreme values*		
**Cohorts**	**NFBC1966 (n=4505)**	**NFBC1986 (n=1010)**	**YFS (n=474)**
2-Hydroxyisobutyrate	3	2	1
Valine	9	0	2
Alanine	2	3	1
Pseudouridine	15	10	0
Glucose	74	5	16
Dimethylamine	48	14	1
Glycine	4	0	0
Citrate	0	0	0
Urea	1	0	0
Formate	13	3	1
Trigonelline	0	0	0
Hippurate	0	0	0
Creatinine	0	0	0

*Associations between the urinary metabolites and body mass index as well as smoking were analysed via linear regression analyses. Extreme metabolite levels (metabolites > third quartile + 8 * interquartile range) were truncated to the values of the upper bound and the metabolite concentrations were log-transformed.

Abbreviations: NFBC, Northern Finland Birth Cohort; YFS, Cardiovascular Risk in Young Finns Study.

Supplementary figures

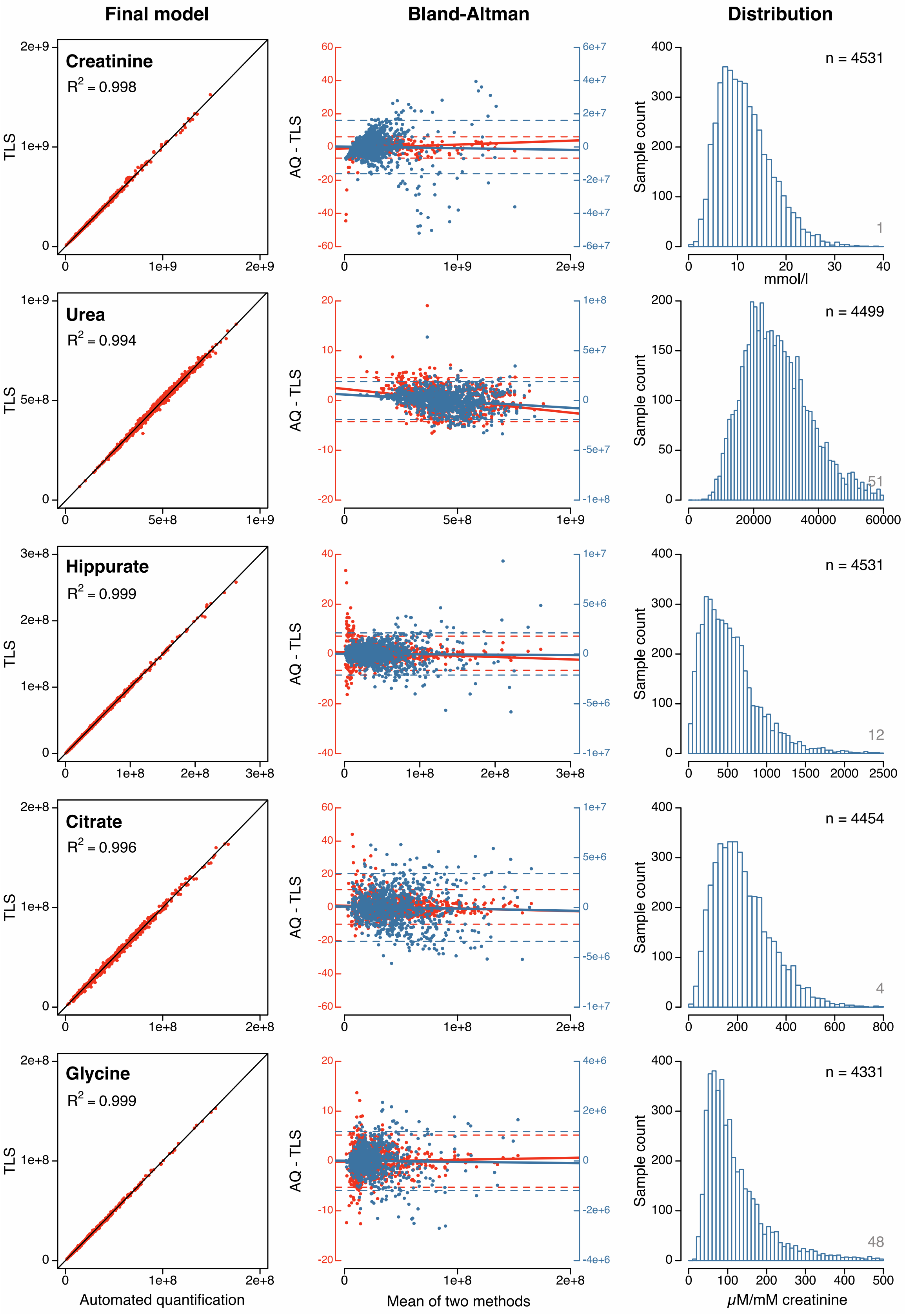

Continued:

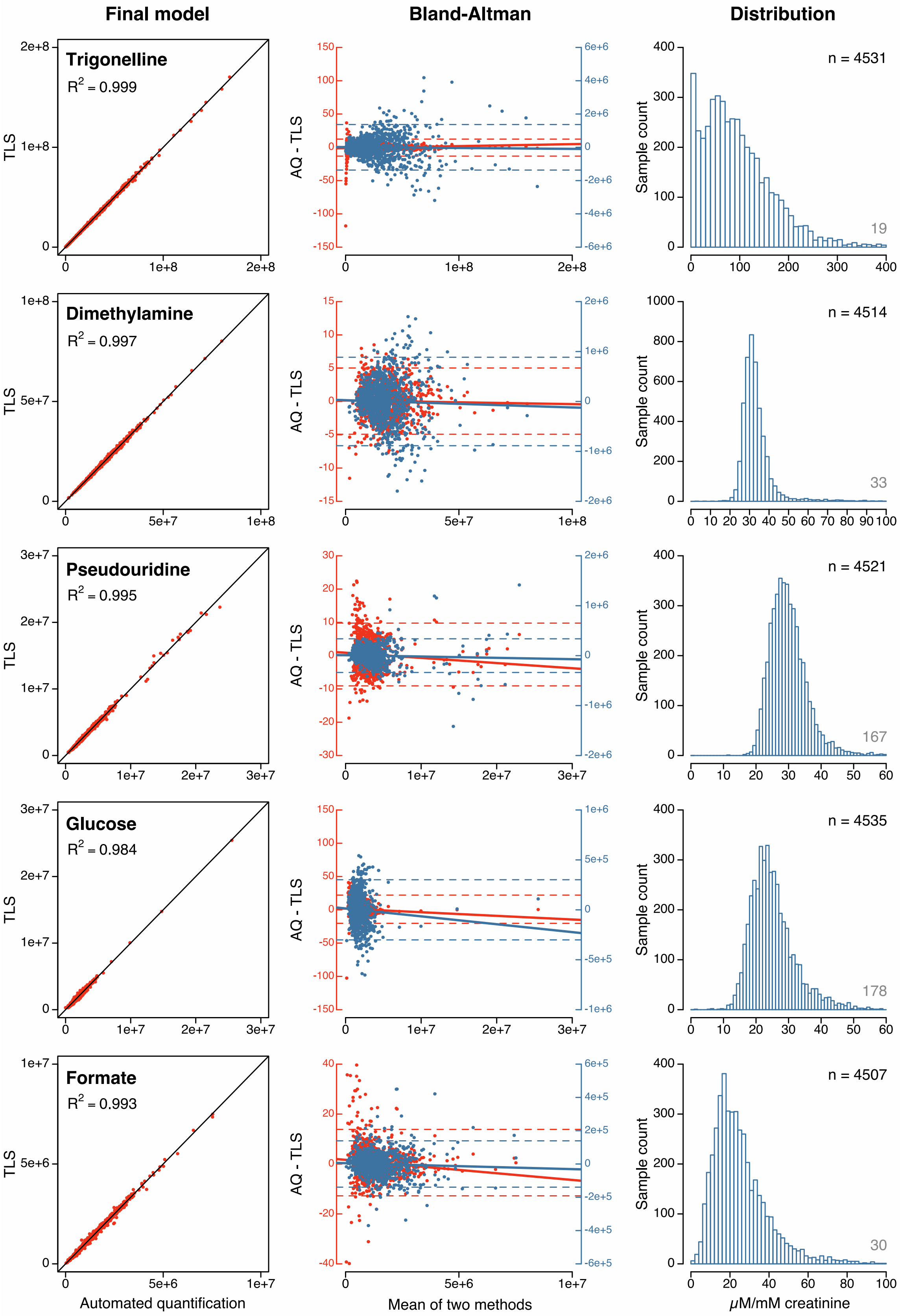
Final model
Bland-Altman
Distribution
Trigonelline
R² = 0.999
n = 4531
19
Dimethylamine
R² = 0.997
n = 4514
33
Pseudouridine
R² = 0.995
n = 4521
167
Glucose
R² = 0.984
n = 4535
178
Formate
R² = 0.993
n = 4507
30
TLS
AQ - TLS
Sample count
Automated quantification
Mean of two methods
μM/mM creatinine

Continued:

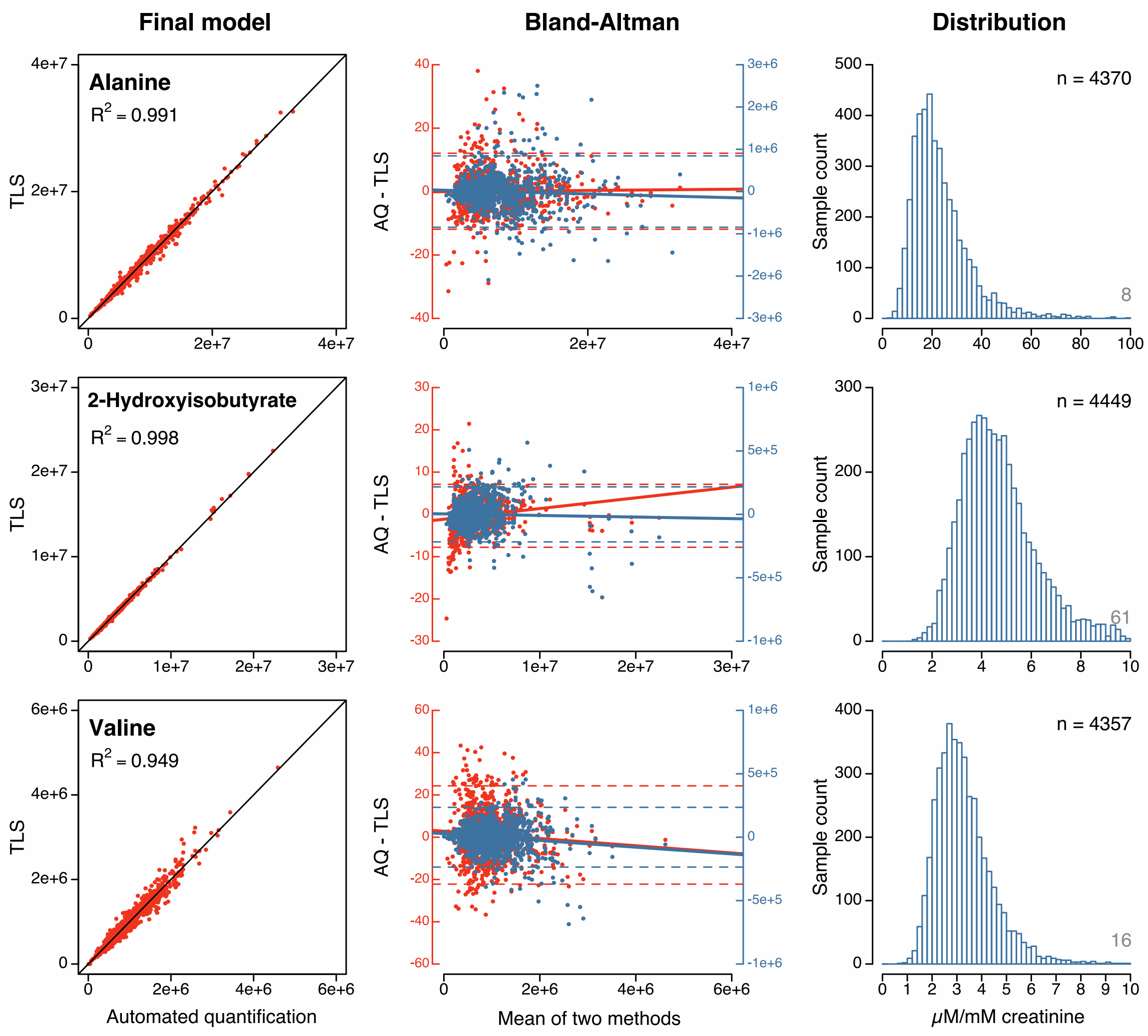

Figure S1. Illustration of the automated quantification models for 13 urinary metabolites. The construction of the automated quantification (AQ) process for urinary metabolites from the nuclear magnetic resonance spectra involves two parts: an alignment and pre-processing of the spectral data and training & validation of a linear regression model for the metabolite concentrations. The alignment and pre-processing include the identification and selection of the metabolite resonance(s) and finetuning the optimal spectral data for quantification. The model training and validation utilises the finetuned spectral data and generalises the implementation of the automated quantification model. The automated quantification analyses were performed with R by developing Bayesian regression-based models using metabolite signal areas determined from ca. 1000 urine spectra from the Northern Finland Birth Cohort (NFBC) 1966 dataset by total lineshape (TLS) fitting analyses as a training set. Validations

were done using first 70-30% training-test splits and then 1000 iteration bootstrapping with 50-50% test-training splits. Samples outside cook's distance and with high leverage were omitted. Convergence must be maintained in both validation procedures, and the final model was trained using the set of samples that maintained the convergence in both validation procedures, thus ensuring robust models. The signal areas obtained by the TLS analyses and the automated quantification models and their squared Pearson's correlations are shown on the left. All metabolites, except urea, pseudouridine, and glucose, were analysed by a single linear regression model. Urea yields some 100 times wider signal compared to most other urinary metabolites. This results in quite a noisy signal, challenging to analyse via automated regression modelling. Thus, automated Lorentzian linefitting algorithm was developed and applied for urea – due to the characteristic broad shape of the resonance, this type of approach worked very well. Pseudouridine has two distinct signals in the urine proton nuclear magnetic resonance spectra. Sometimes other metabolite signals appear and overlap with the primary pseudouridine signal (at around 7.677 ppm); in these cases, the secondary signal (at around 4.69 ppm) is used. Both signal areas have their own specific regression model. In the case of glucose linear regression modelling was applied in the normoglycemic concentration range and numerical integration for very large signals. The glucose resonance tends to be wider for very high urinary glucose concentrations and thus a single regression model did not work in an optimal way. The middle row depicts the Bland-Altman plots including percentual (red line) and absolute (blue line) bias between the signal areas obtained by AQ and TLS. Distributions of the corresponding metabolite concentrations (μM/mM creatinine) for the entire NFBC1966 are shown on the right. The grey numbers refer to very high concentrations that are omitted from the graphs for clarity.

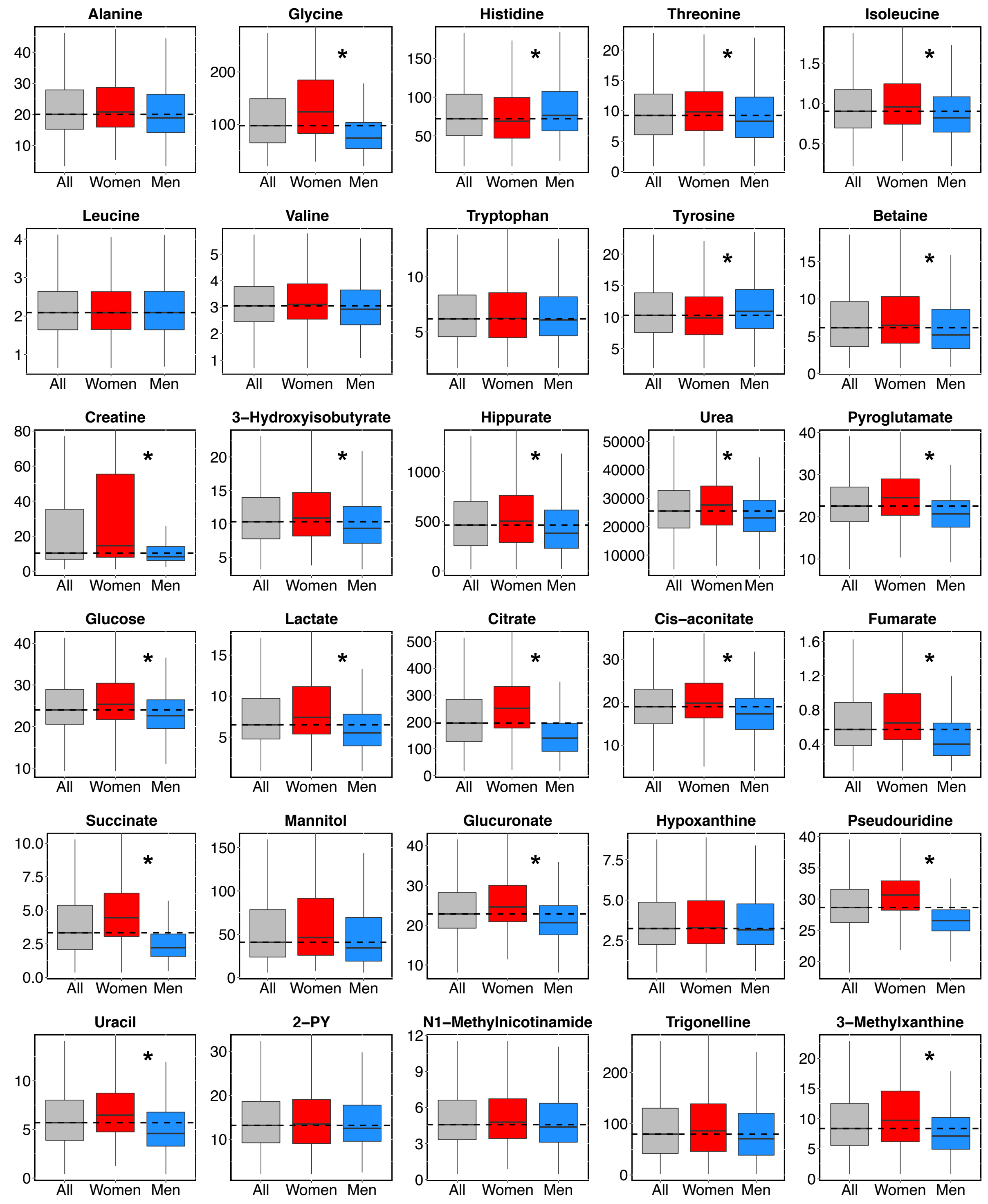
Alanine
Glycine
Histidine
Threonine
Isoleucine
Leucine
Valine
Tryptophan
Tyrosine
Betaine
Creatine
3–Hydroxyisobutyrate
Hippurate
Urea
Pyroglutamate
Glucose
Lactate
Citrate
Cis–aconitate
Fumarate
Succinate
Mannitol
Glucuronate
Hypoxanthine
Pseudouridine
Uracil
2–PY
N1–Methylnicotinamide
Trigonelline
3–Methylxanthine
All
Women
Men

Continued:

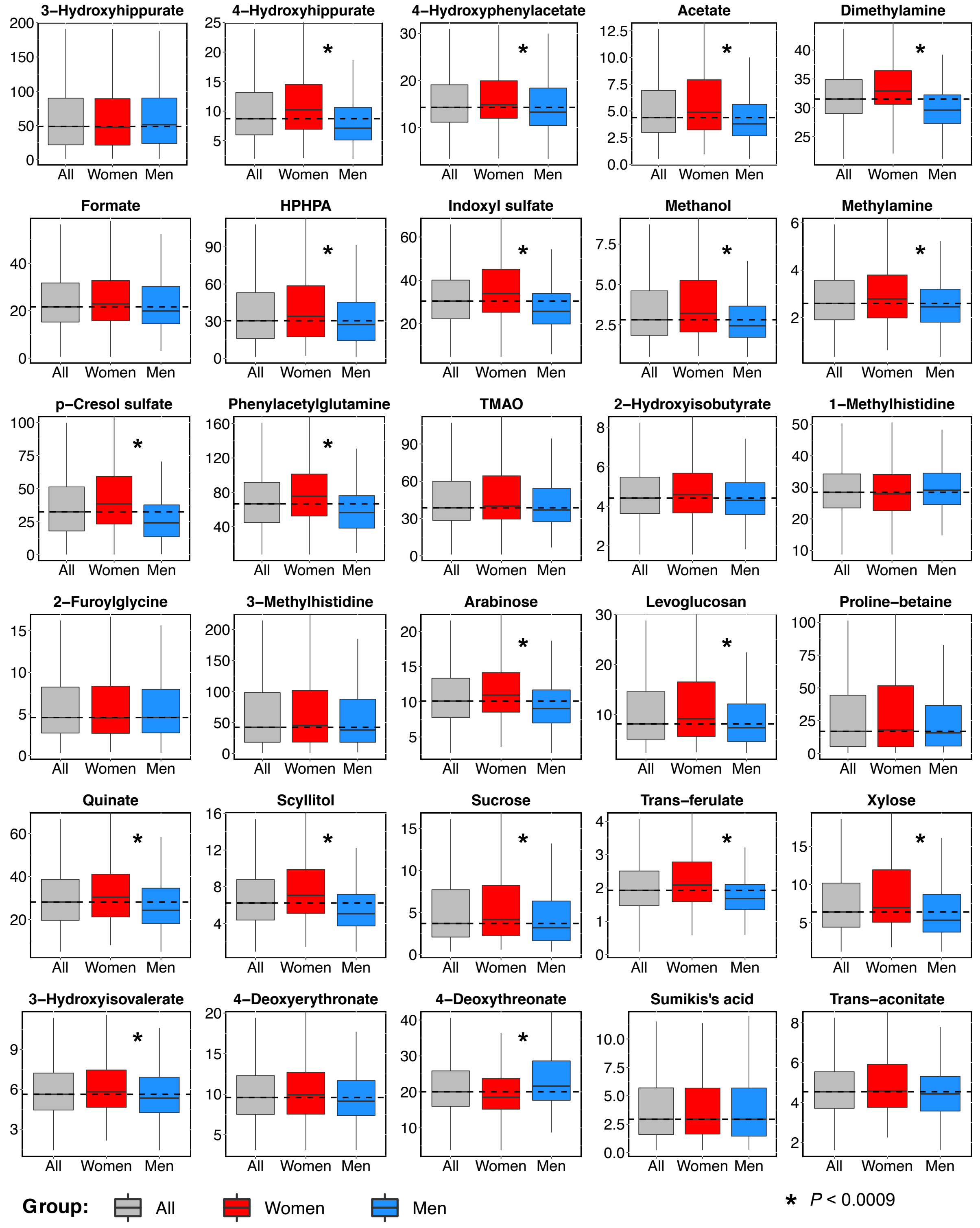

Figure S2. Boxplots of 60 quantified urine metabolites (referenced to creatinine, µM/mM creatinine) in all individuals (n=994), men (n=419), and women (n=575) in the random subset of Northern Finland Birth Cohort 1966. The horizonal dashed lines indicate the median of each metabolite in all individuals. * $P < 0.0009$ (Wilcoxon's Signed Rank Test for the comparisons between men and women). 2-PY, N1-Methyl-2-pyridone-5-carboxamide; HPHPA, 3-(3-Hydroxyphenyl)-3-hydroxypropanoate; TMAO, trimethylamine N-oxide.

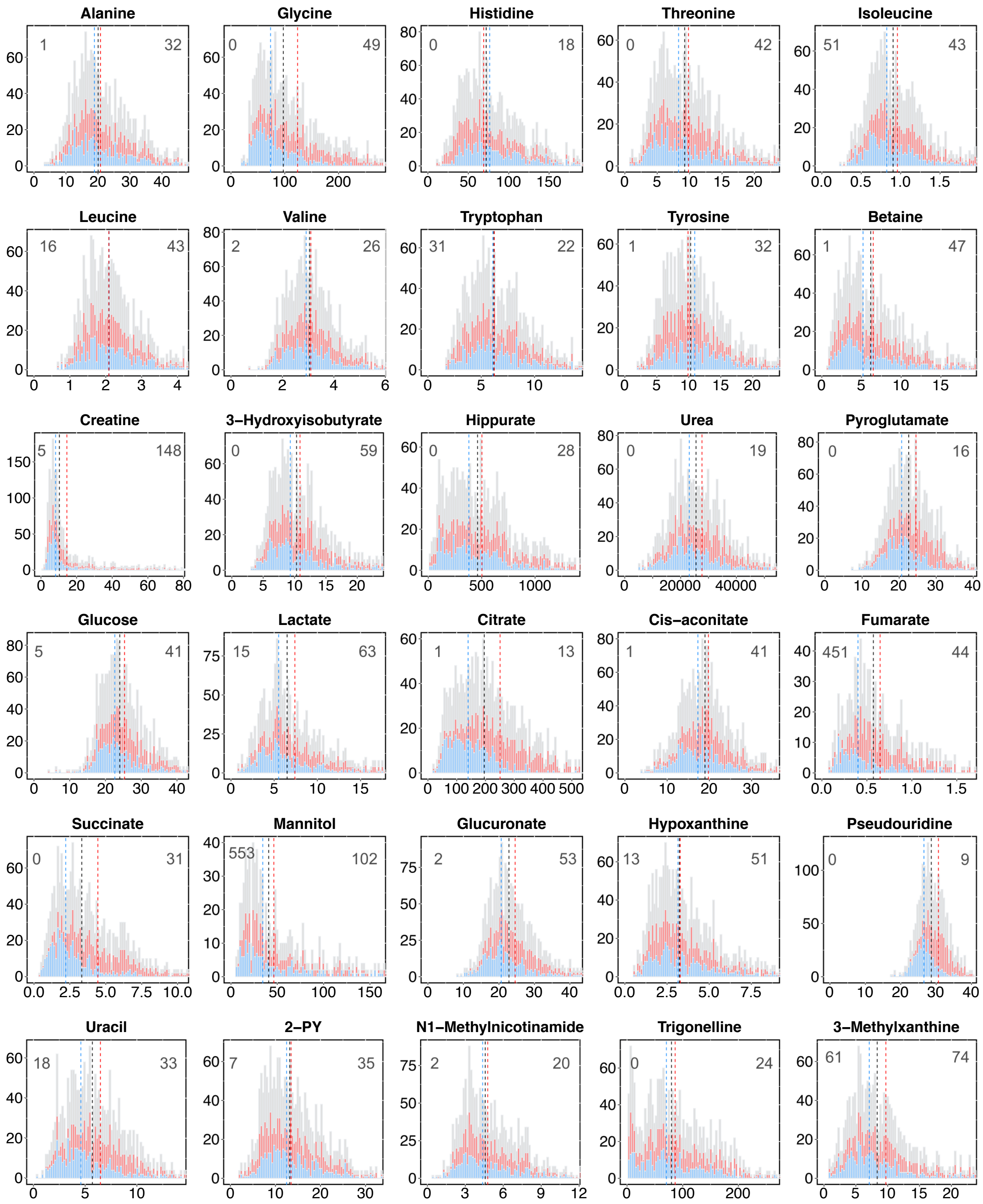

Alanine 1 32
Glycine 0 49
Histidine 0 18
Threonine 0 42
Isoleucine 51 43
Leucine 16 43
Valine 2 26
Tryptophan 31 22
Tyrosine 1 32
Betaine 1 47
Creatine 5 148
3–Hydroxyisobutyrate 0 59
Hippurate 0 28
Urea 0 19
Pyroglutamate 0 16
Glucose 5 41
Lactate 15 63
Citrate 1 13
Cis–aconitate 1 41
Fumarate 451 44
Succinate 0 31
Mannitol 553 102
Glucuronate 2 53
Hypoxanthine 13 51
Pseudouridine 0 9
Uracil 18 33
2–PY 7 35
N1–Methylnicotinamide 2 20
Trigonelline 0 24
3–Methylxanthine 61 74

Continued:

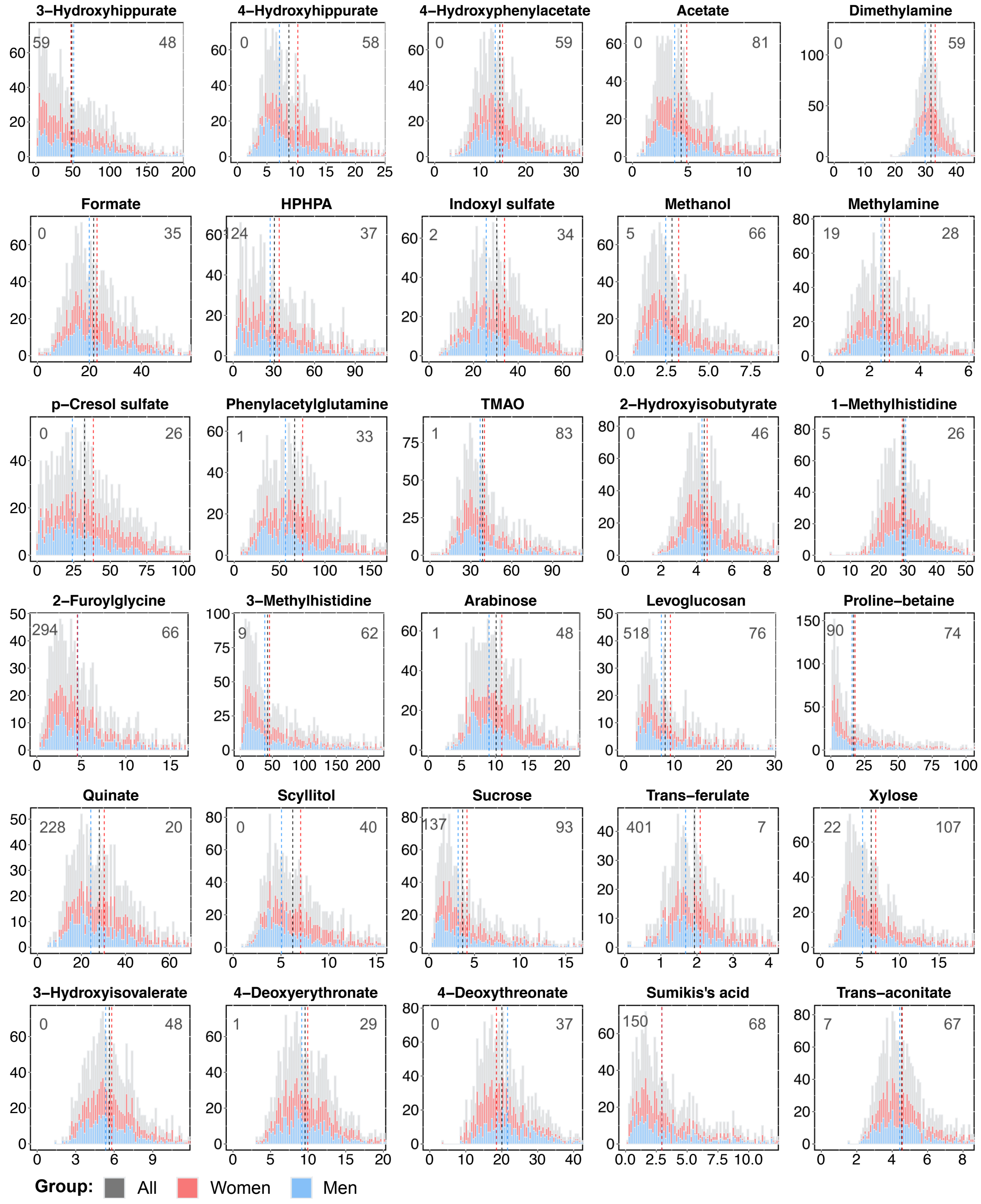

Figure S3. Histograms of 60 quantified urine metabolites (referenced to creatinine, µM/mM creatinine) in all individuals (n=994), men (n=419), and women (n=575) in the random subset of Northern Finland Birth Cohort 1966. The vertical dashed lines indicate the median of each metabolite in all individuals (black), men (blue), and women (red). The number in the top left corner refers to the number of zero values for each metabolite and the number in the top right corner to the number of high concentration values not shown in the histograms for visual clarity. 2-PY, N1-Methyl-2-pyridone-5-carboxamide; HPHPA, 3-(3-Hydroxyphenyl)-3-hydroxypropanoate; TMAO, trimethylamine N-oxide.

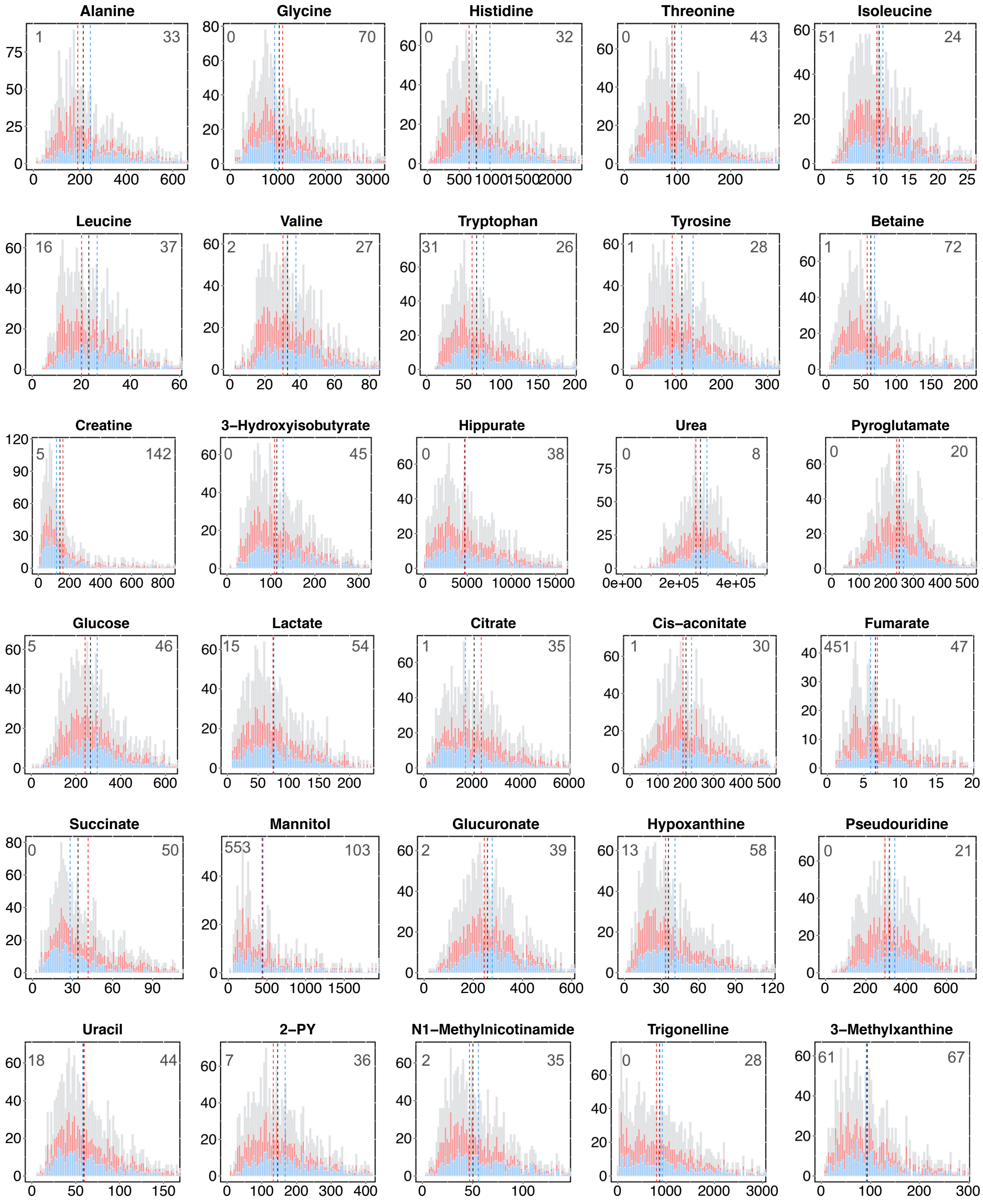
Alanine
Glycine
Histidine
Threonine
Isoleucine
Leucine
Valine
Tryptophan
Tyrosine
Betaine
Creatine
3–Hydroxyisobutyrate
Hippurate
Urea
Pyroglutamate
Glucose
Lactate
Citrate
Cis–aconitate
Fumarate
Succinate
Mannitol
Glucuronate
Hypoxanthine
Pseudouridine
Uracil
2–PY
N1–Methylnicotinamide
Trigonelline
3–Methylxanthine

Continued:

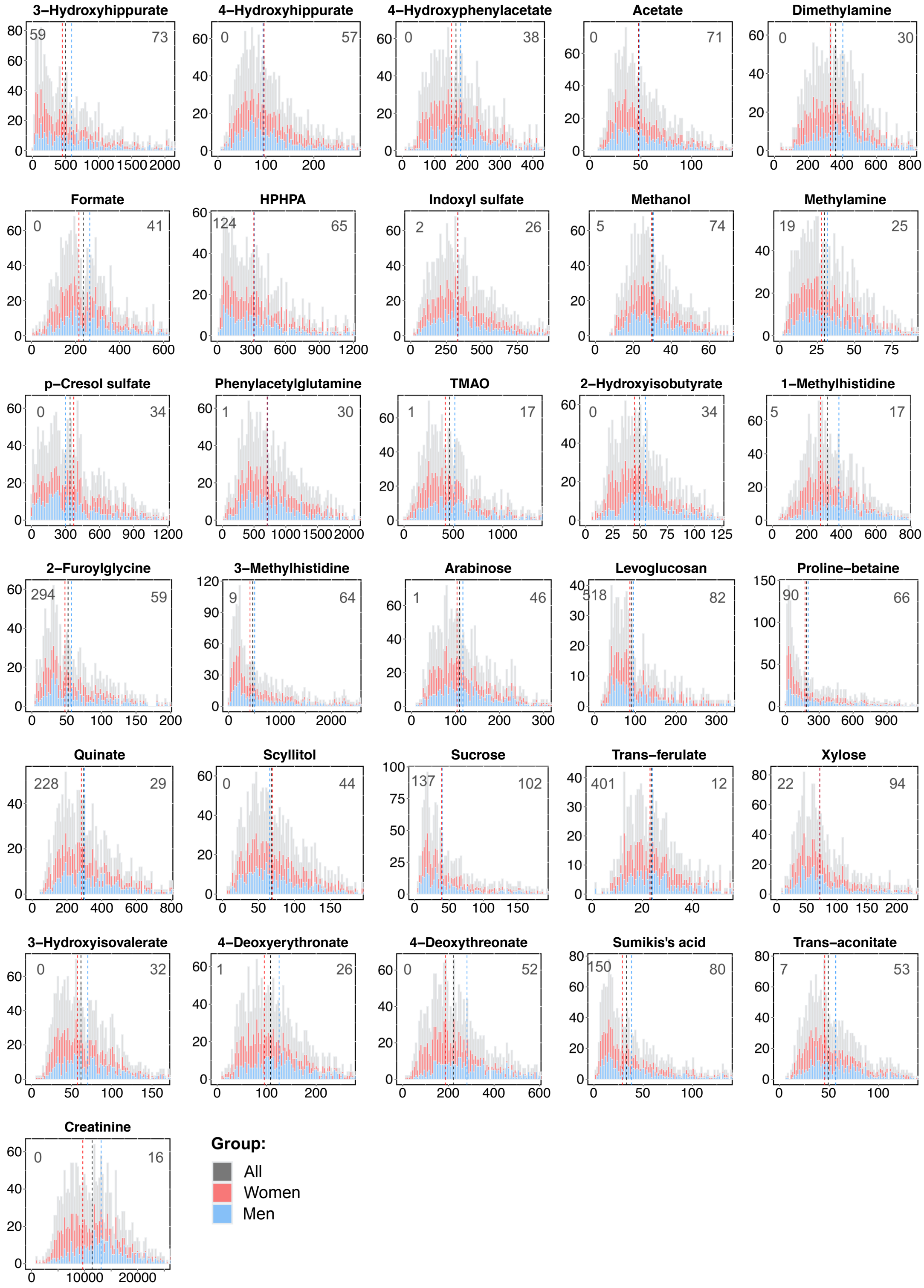

Figure S4. Histograms of 61 quantified urine metabolites (absolute concentrations, µmol/L) in all individuals (n=994), men (n=419), and women (n=575) in the random subset of Northern Finland Birth Cohort 1966. The vertical dashed lines indicate the median of each metabolite in all individuals (black), men (blue), and women (red). The number in the top left corner refers to the number of zero values for each metabolite and the number in the top right corner to the number of high concentration values not shown in the histograms for visual clarity. 2-PY, N1-Methyl-2-pyridone-5-carboxamide; HPHPA, 3-(3-Hydroxyphenyl)-3-hydroxypropanoate; TMAO, trimethylamine N-oxide.

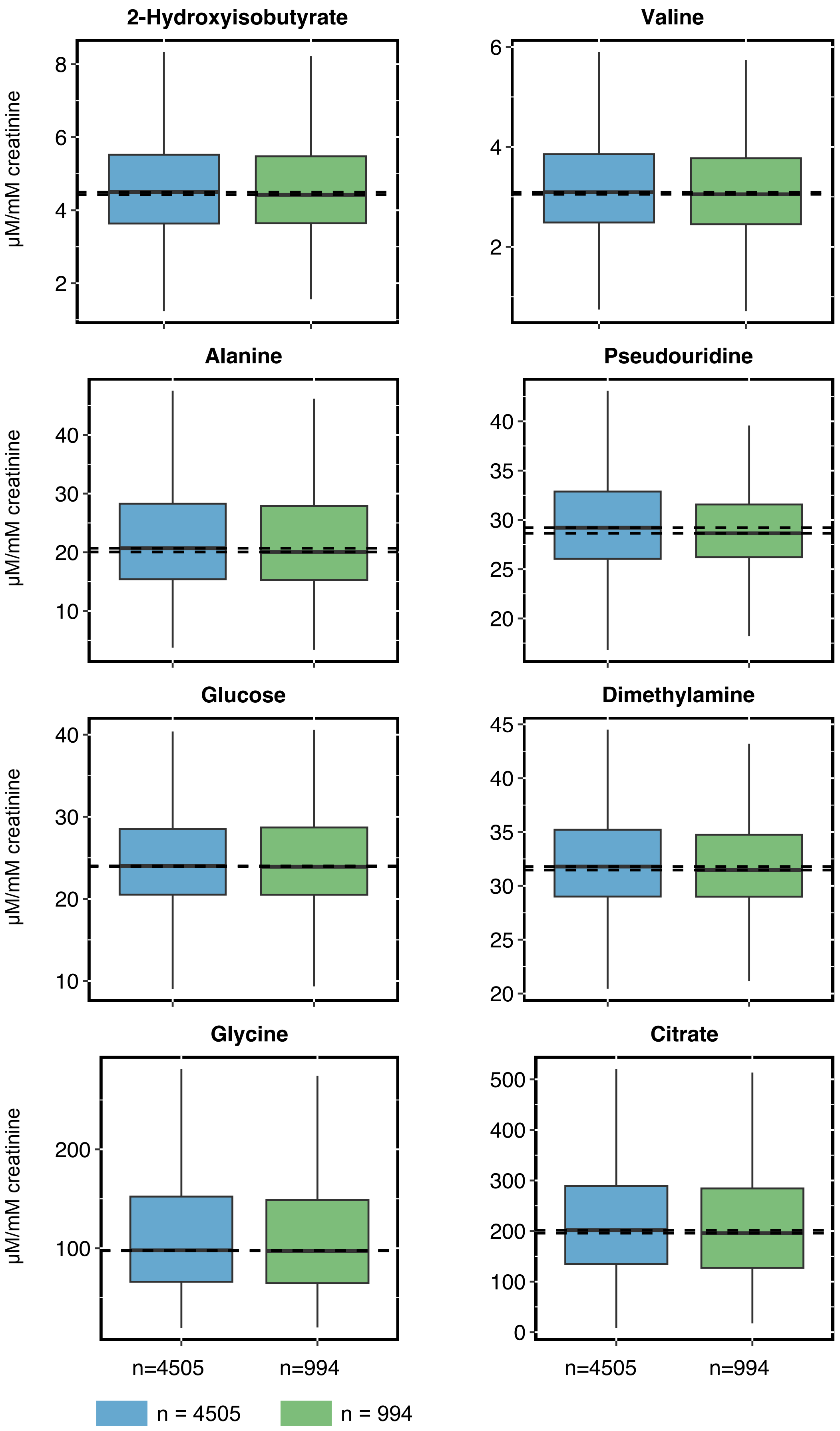
2-Hydroxyisobutyrate
Valine
Alanine
Pseudouridine
Glucose
Dimethylamine
Glycine
Citrate
μM/mM creatinine
n=4505
n=994
n = 4505
n = 994

Continued:

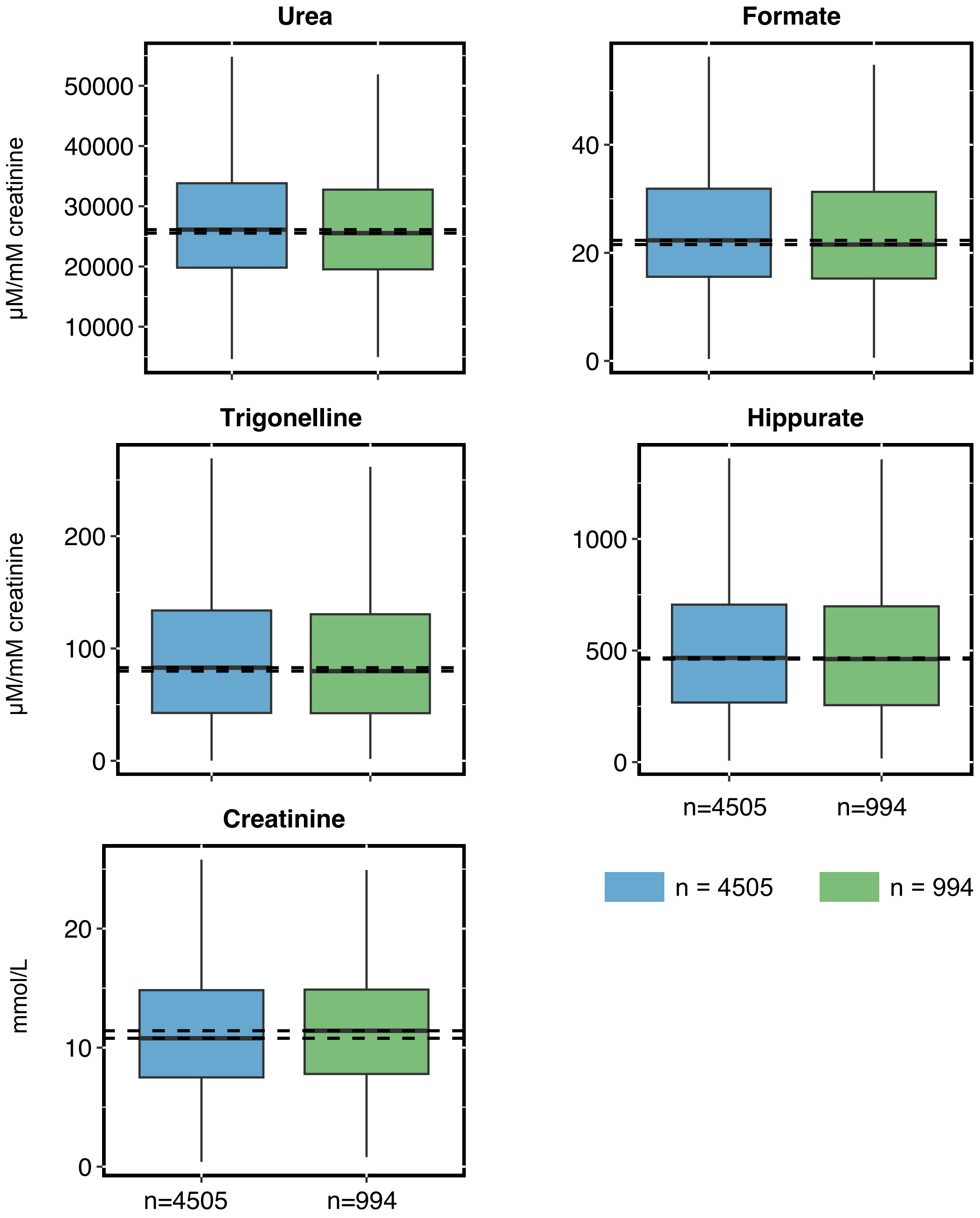

Figure S5. Boxplots of the 13 automatically quantified urinary metabolites in all the Northern Finland Birth Cohort 1966 participants (n=4505) and in the random subset (n=994). The horizonal dashed lines are drawn to facilitate comparisons between the medians. The unit for the concentrations is µM/mM creatinine, except for creatinine for which it is mmol/L. * $P < 0.0009$ (Wilcoxon's Signed Rank Test for the comparisons).

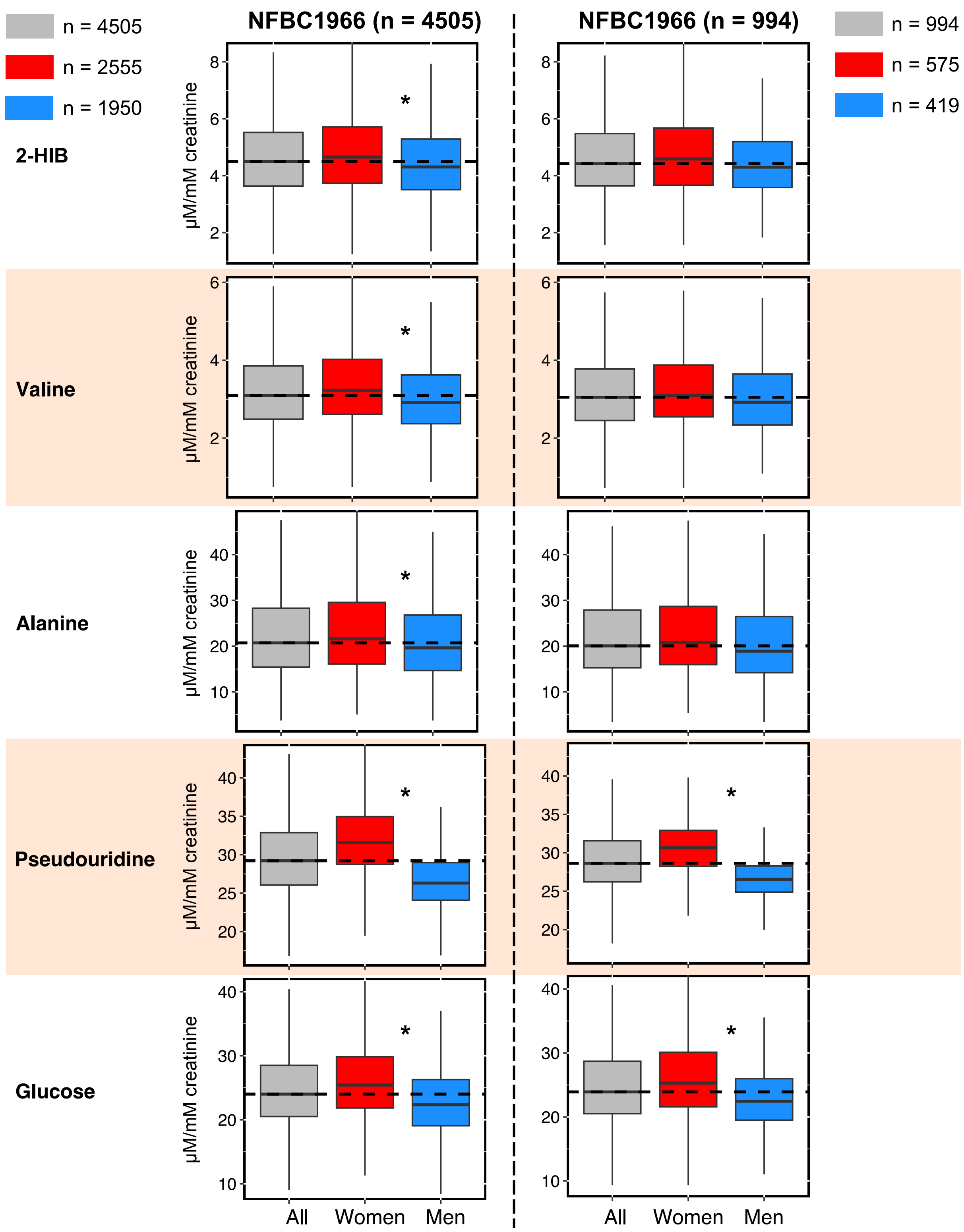

n = 4505
n = 2555
n = 1950
2-HIB
NFBC1966 (n = 4505)
NFBC1966 (n = 994)
n = 994
n = 575
n = 419
Valine
Alanine
Pseudouridine
Glucose
µM/mM creatinine
*
All
Women
Men

Continued:

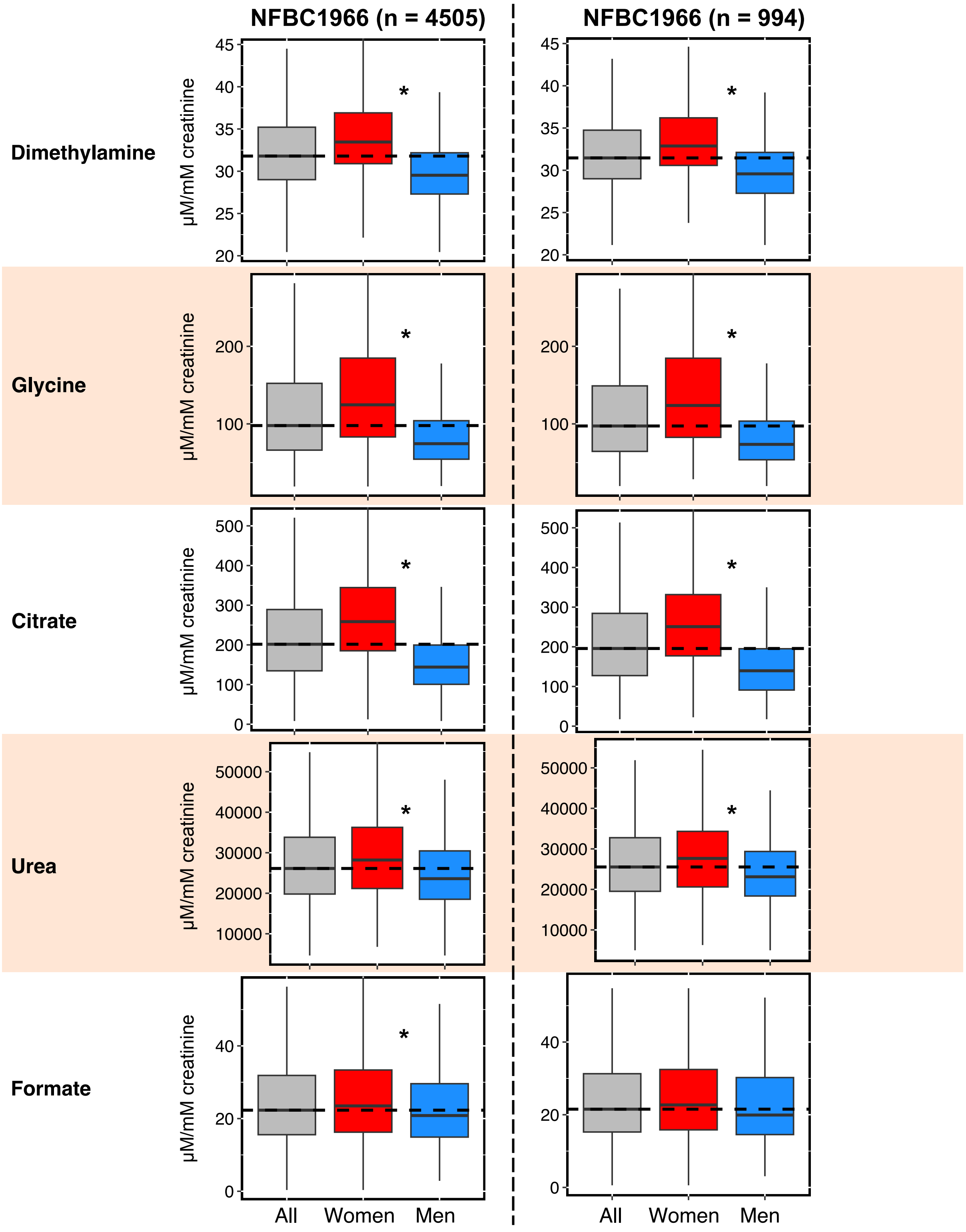
NFBC1966 (n = 4505)
NFBC1966 (n = 994)
Dimethylamine
Glycine
Citrate
Urea
Formate
µM/mM creatinine
*
All
Women
Men

Continued:

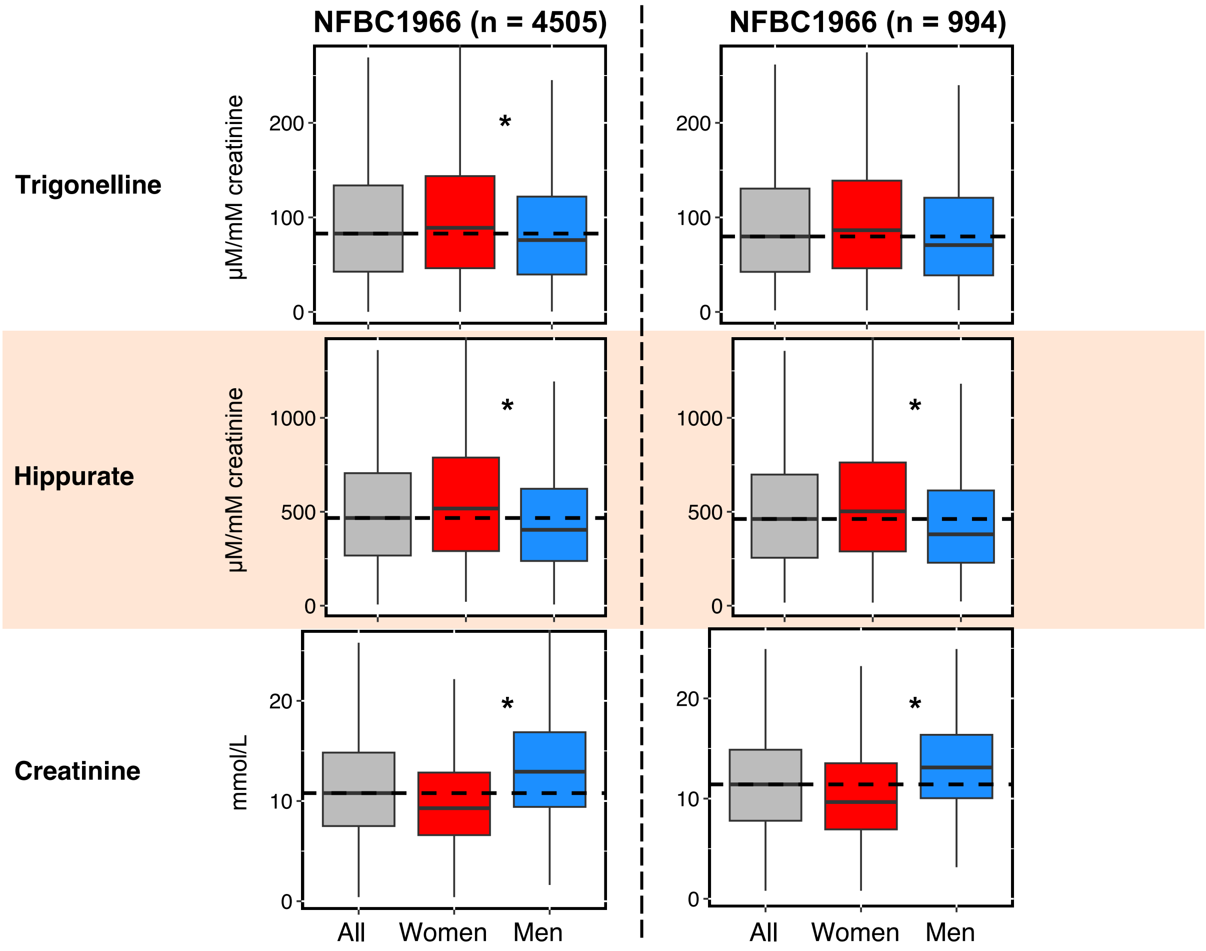

Figure S6. Boxplots of 13 automatically quantified urinary metabolites in the Northern Finland Birth Cohort 1966. In the entire cohort (n=4505) there are 1950 men and 2555 women and in the random subset (n=994) 419 men and 575 women. The horizonal dashed lines indicate the median of each metabolite in all individuals, i.e., in 4505 participants for the entire cohort (on the left) and in 994 individuals for the random subset (on the right). The unit for the concentrations is µM/mM creatinine, except for creatinine for which it is mmol/L. * $P < 0.0009$ (Wilcoxon's Signed Rank Test for the comparisons). 2-HIB: 2-Hydroxyisobutyrate; NFBC, Northern Finland Birth Cohort.

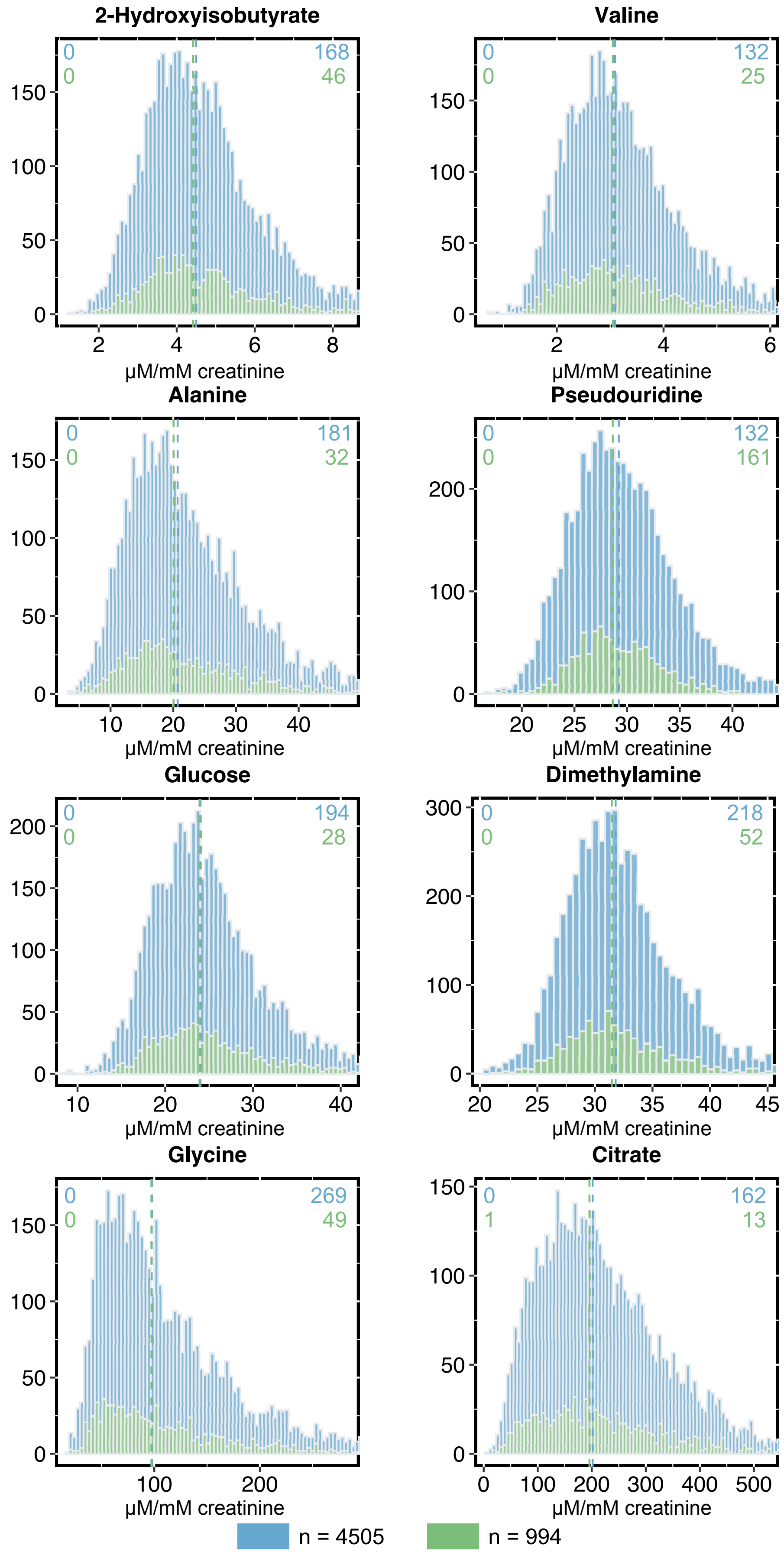
2-Hydroxyisobutyrate
0
0
168
46
μM/mM creatinine
Valine
0
0
132
25
μM/mM creatinine
Alanine
0
0
181
32
μM/mM creatinine
Pseudouridine
0
0
132
161
μM/mM creatinine
Glucose
0
0
194
28
μM/mM creatinine
Dimethylamine
0
0
218
52
μM/mM creatinine
Glycine
0
0
269
49
μM/mM creatinine
Citrate
0
1
162
13
μM/mM creatinine
n = 4505
n = 994

Continued:

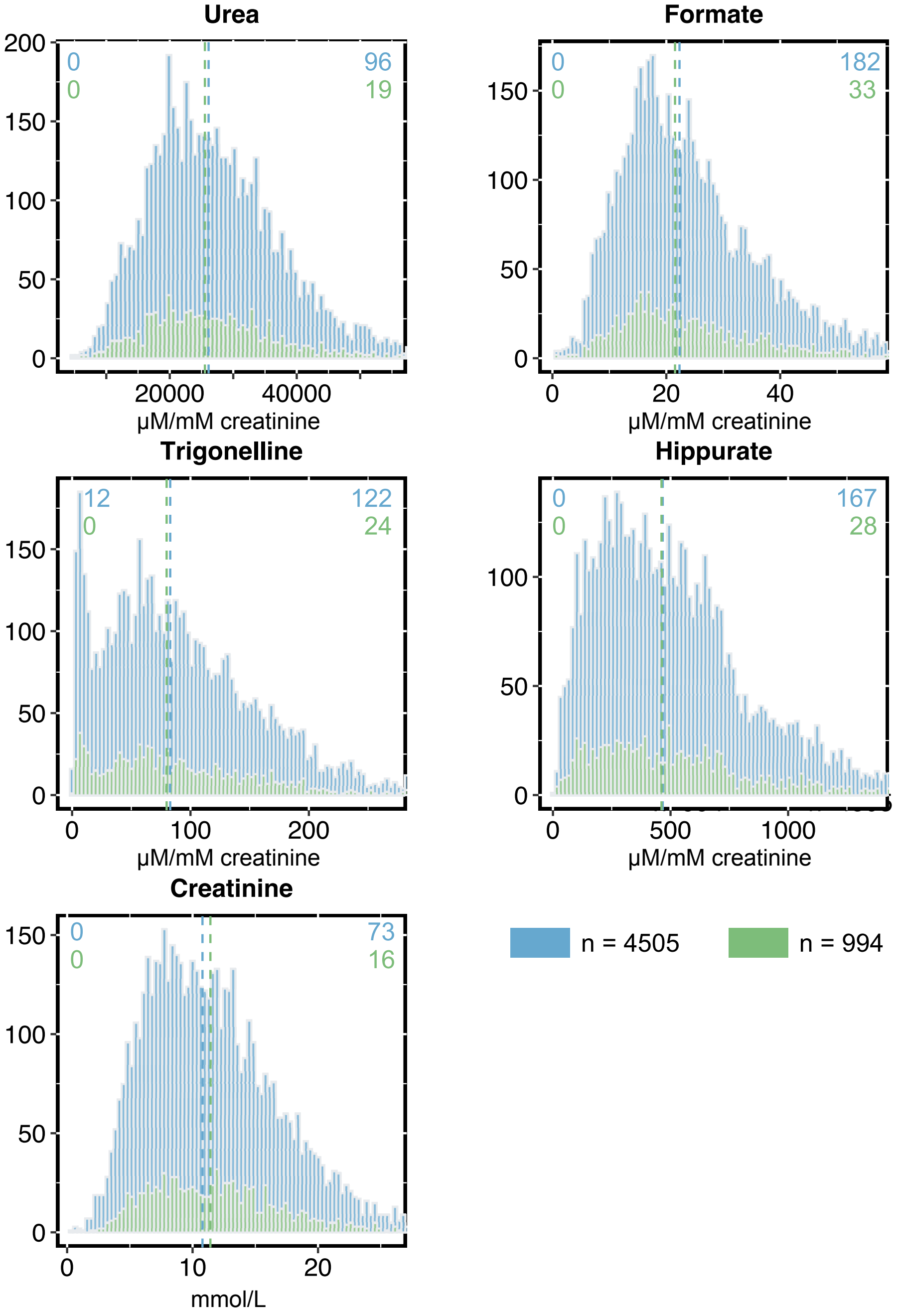

Figure S7. Histograms of 13 automatically quantified urinary metabolites in all the Northern Finland Birth Cohort 1966 participants (n=4505; blue) and in the random subset (n=994; green). The unit for the concentrations is µM/mM creatinine, except for creatinine for which it is mmol/L. The vertical dashed lines indicate the median for each metabolite. The number in the top left corner refers to the number of zero values for each metabolite and the number in the top right corner to the number of high concentration values not shown in the histograms for visual clarity; blue numbers refer to all Northern Finland Birth Cohort 1966 participants and the green numbers to the random subset.

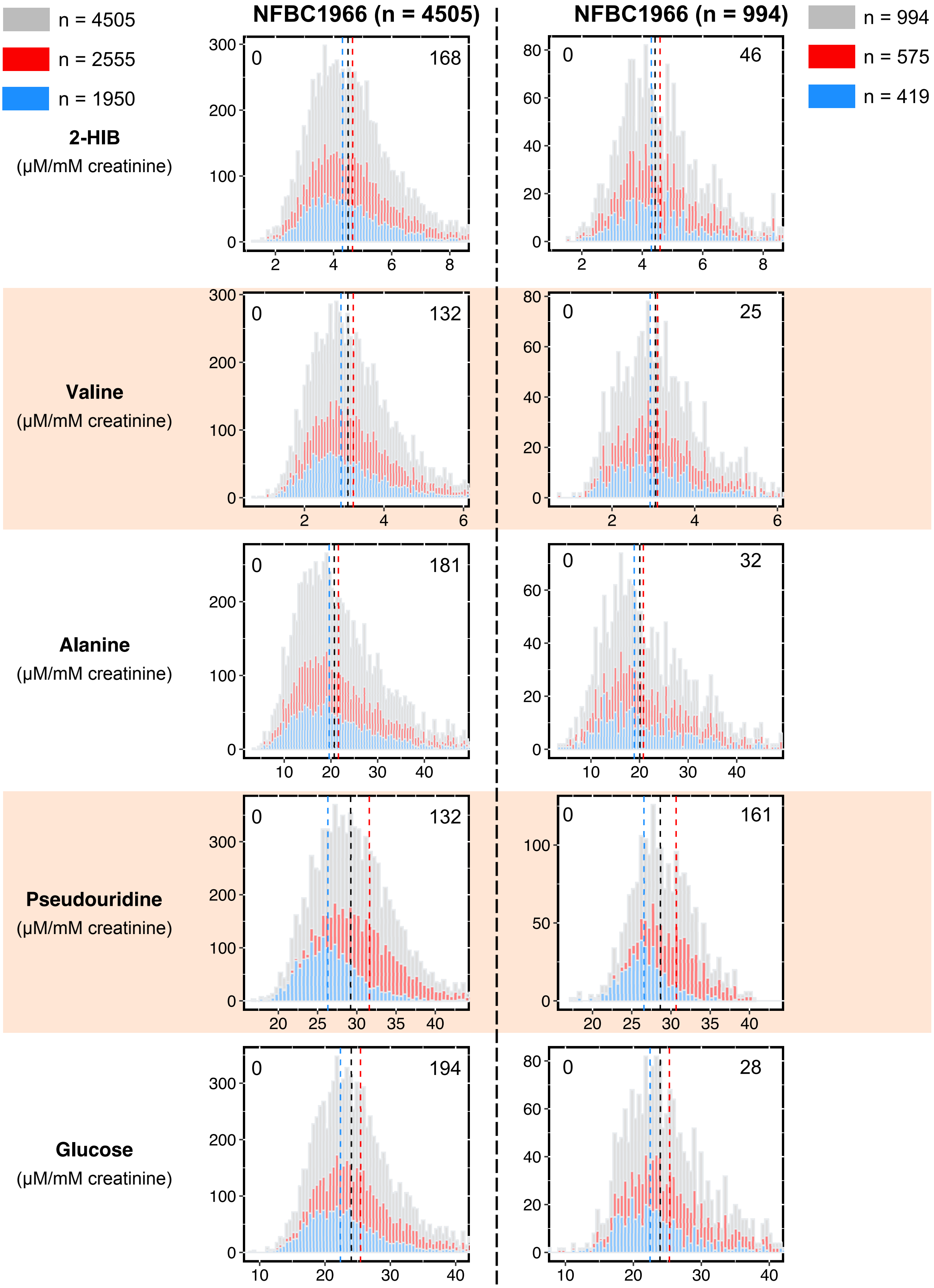
NFBC1966 (n = 4505)
NFBC1966 (n = 994)
n = 4505
n = 2555
n = 1950
n = 994
n = 575
n = 419
2-HIB
(µM/mM creatinine)
Valine
(µM/mM creatinine)
Alanine
(µM/mM creatinine)
Pseudouridine
(µM/mM creatinine)
Glucose
(µM/mM creatinine)
0
168
46
132
25
181
32
132
161
194
28

Continued:

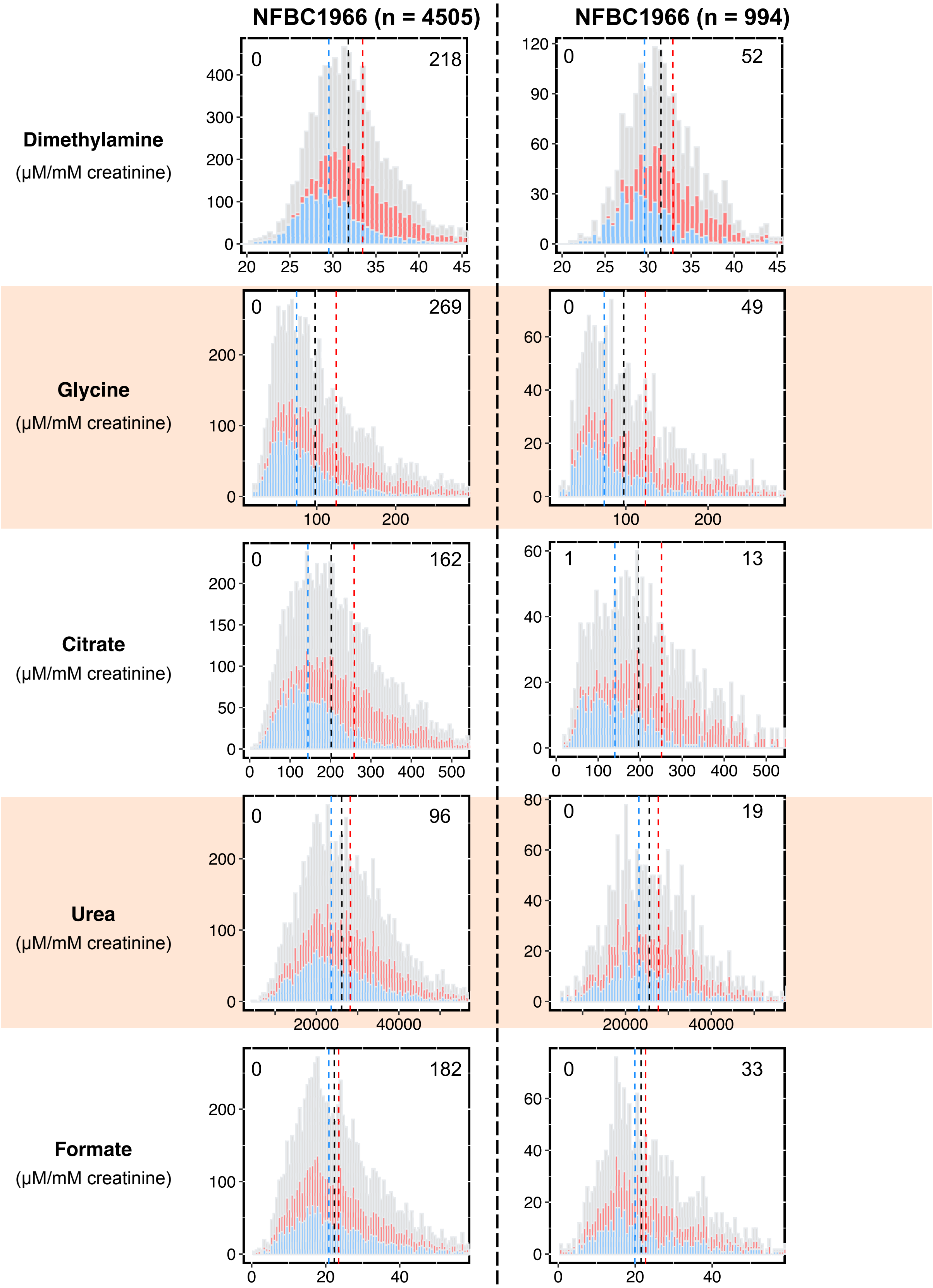
NFBC1966 (n = 4505)
NFBC1966 (n = 994)
Dimethylamine
(μM/mM creatinine)
0
218
0
52
Glycine
(μM/mM creatinine)
0
269
0
49
Citrate
(μM/mM creatinine)
0
162
1
13
Urea
(μM/mM creatinine)
0
96
0
19
Formate
(μM/mM creatinine)
0
182
0
33

Continued:

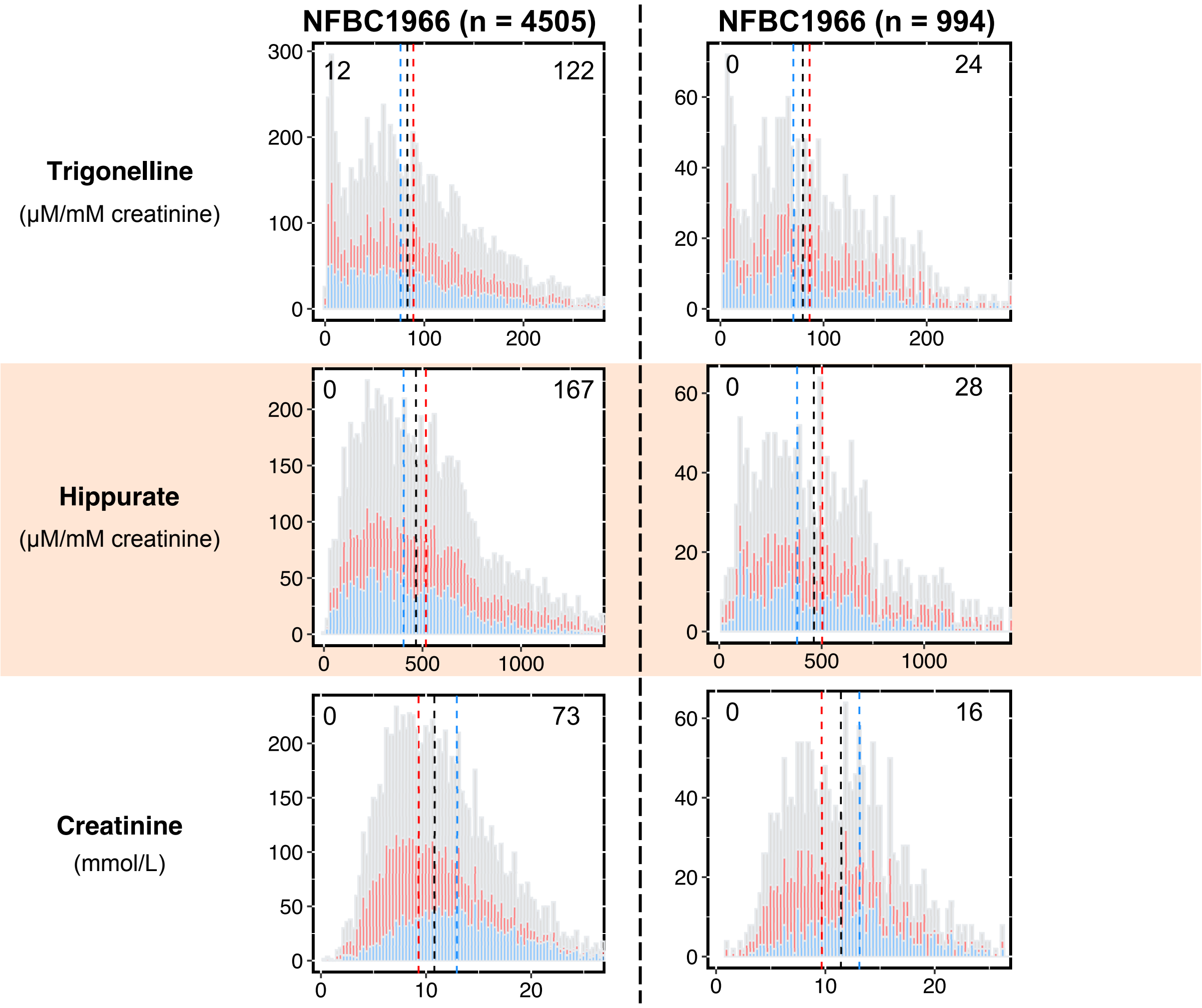

Figure S8. Histograms of 13 automatically quantified urinary metabolites in men and women in all the Northern Finland Birth Cohort 1966 participants (n=4505) and in the random subset (n=994). In the entire cohort (n=4505) there are 1950 men and 2555 women and in the random subset (n=994) 419 men and 575 women. The unit for the concentrations is µM/mM creatinine, except for creatinine for which it is mmol/L. The vertical dashed lines indicate the median of each metabolite in all individuals (black), men (blue), and women (red) in the entire cohort (left) and in the random subset (right). The number in the top left corner refers to the number of zero values for each metabolite and the number in the top right corner to the number of high concentration values not shown in the histograms for visual clarity. The numbers in the left column refer to the entire cohort and those in the right column to the random subset. 2-HIB: 2-Hydroxyisobutyrate; NFBC, Northern Finland Birth Cohort.

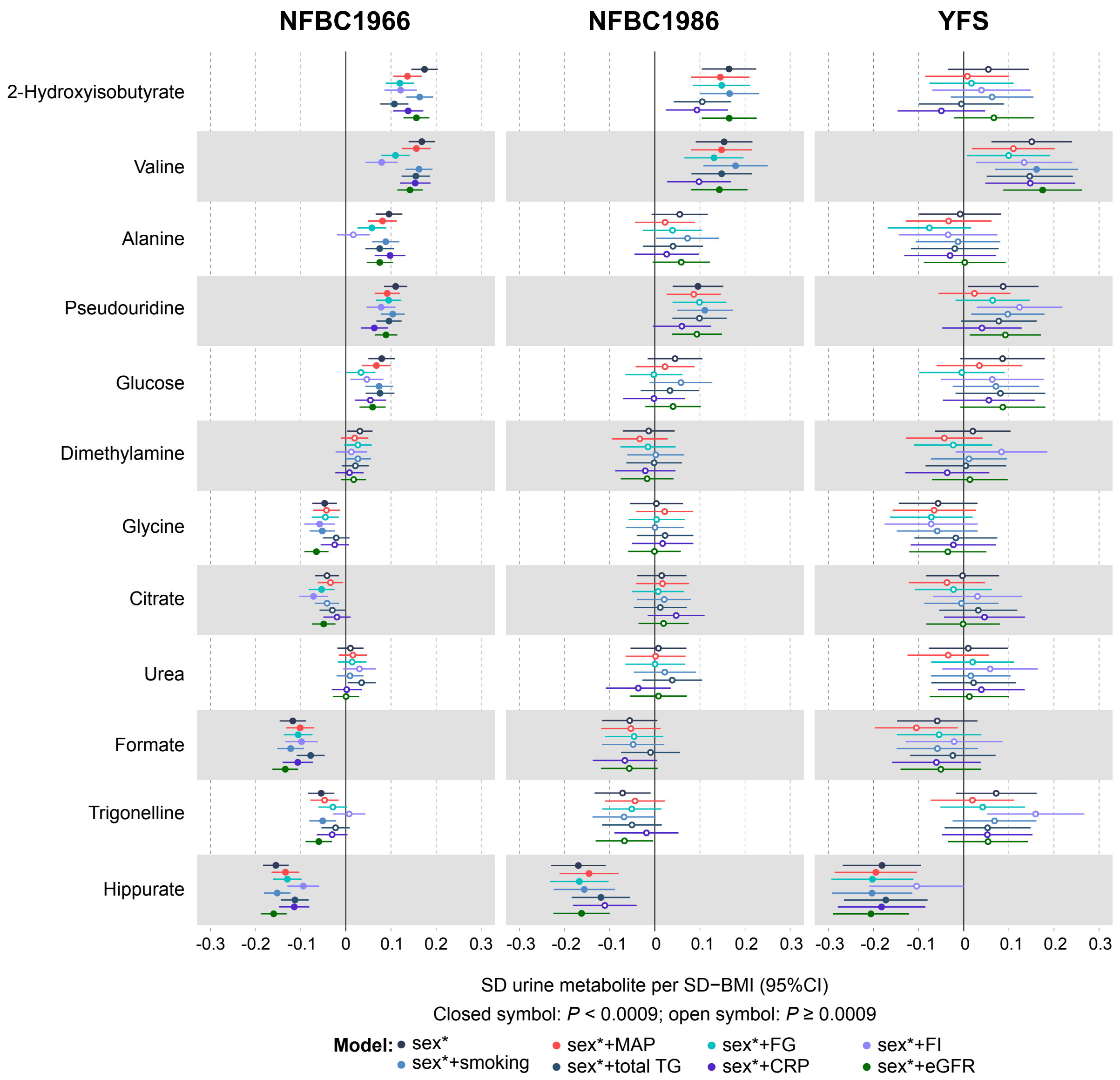

Figure S9. Regression models of 12 automatically quantified urinary metabolites (referenced to creatinine) for BMI in Northern Finland Birth Cohort (NFBC1966) (n=4505), NFBC1986 (n=1010) and Cardiovascular Risk in Young Finns Study (n=474). The base models were adjusted for sex (and age in the YFS) on the top of which seven different adjustments were individually added to assess their potential confounding role: MAP, FG, FI, smoking, TG, CRP, and eGFR. FI was not available for NFBC1986. * denotes that age was also adjusted for YFS. MAP, mean arterial pressure; FG, fasting glucose; FI, fasting insulin; TG, total triglycerides; CRP, C-reactive protein; eGFR, estimated glomerular filtration rate; NFBC, Northern Finland Birth Cohort; YFS, Cardiovascular Risk in Young Finns Study.

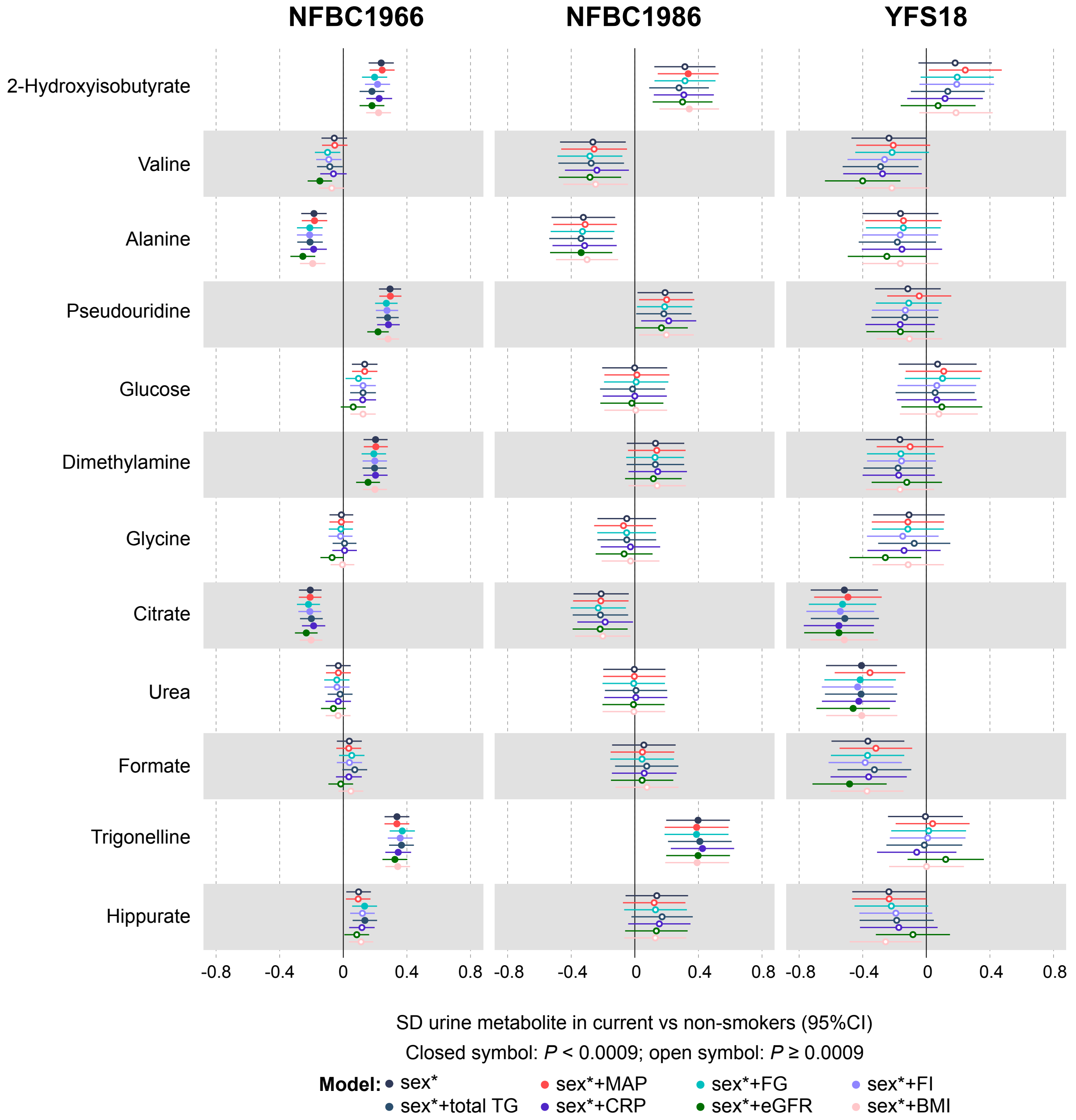

Figure S10. Regression models of 12 automatically quantified urinary metabolites (referenced to creatinine) for smoking in Northern Finland Birth Cohort 1966 (n=4505), NFBC1986 (n=1010) and Cardiovascular Risk in Young Finns Study (n=474). The base models were adjusted for sex (and age in the Cardiovascular Risk in Young Finns Study (YFS)) on the top of which seven different adjustments were individually added to assess their potential confounding role: MAP, FG, FI, TG, CRP, eGFR, and BMI. The smoking data for the cohorts are: Northern Finland Birth Cohort (NFBC) 1966, 750 current and 3544 non-smokers; NFBC1986, 115 current and 706 non-smokers; and YFS, 85 current and 370 non-smokers. FI was not available for NFBC1986. * denotes that age was also adjusted for YFS. MAP, mean arterial pressure; FG, fasting glucose; FI, fasting insulin; TG, total triglycerides; CRP, C-reactive protein; eGFR, estimated glomerular filtration rate; BMI, body mass index; NFBC, Northern Finland Birth Cohort; YFS, Cardiovascular Risk in Young Finns Study.

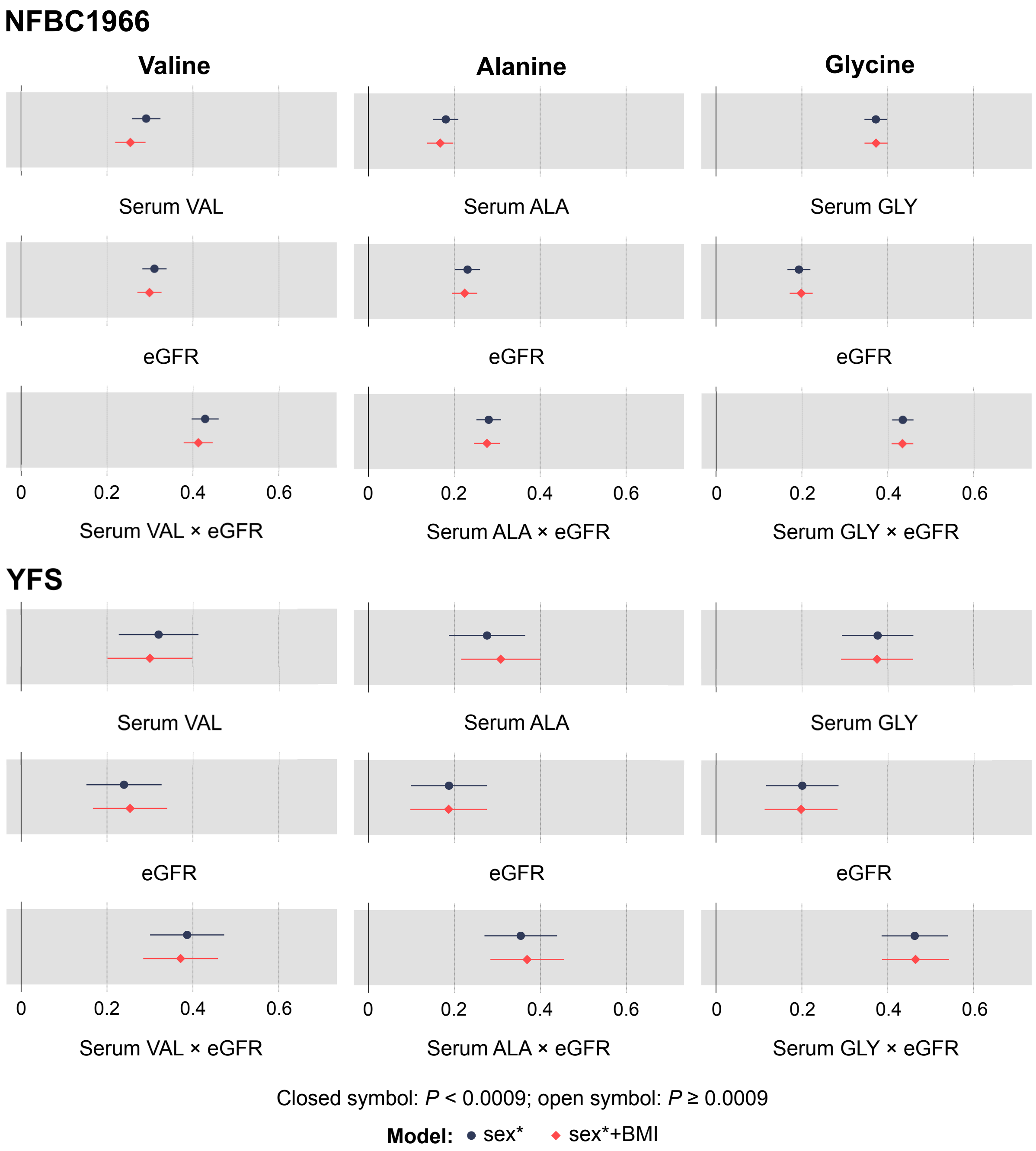

Figure S11. Regression models of three automatically quantified urinary amino acid (valine, alanine, and glycine) concentrations (referenced to creatinine) and their corresponding serum concentrations, estimated glomerular filtration rate (eGFR), and the multiplication of the serum concentration and eGFR in Northern Finland Birth Cohort 1966 (n=4505) and Cardiovascular Risk in Young Finns Study (n=474). The effects of sex (black) and sex + BMI (red) were examined; * that age was also adjusted for YFS. eGFR, estimated glomerular filtration rate; NFBC, Northern Finland Birth Cohort; YFS, Cardiovascular Risk in Young Finns Study.

References

[1] T. Nordström *et al.*, “Cohort Profile: 46 years of follow-up of the Northern Finland Birth Cohort 1966 (NFBC1966),” *Int. J. Epidemiol.*, vol. 50, no. 6, pp. 1786-1787j, 2022.

[2] O. T. Raitakari *et al.*, “Cohort Profile: The Cardiovascular Risk in Young Finns Study,” *Int. J. Epidemiol.*, vol. 37, no. 6, pp. 1220–1226, Dec. 2008.

[3] P. Soininen *et al.*, “High-throughput serum NMR metabonomics for cost-effective holistic studies on systemic metabolism.,” *Analyst*, vol. 134, no. 9, pp. 1781–5, Sep. 2009.

[4] L. A. Inker *et al.*, “Estimating glomerular filtration rate from serum creatinine and cystatin C.,” *N. Engl. J. Med.*, vol. 367, no. 1, pp. 20–9, Jul. 2012.

[5] R. Bhopal, C. Fischbacher, E. Vartiainen, N. Unwin, M. White, and G. Alberti, “Predicted and observed cardiovascular disease in South Asians: Application of FINRISK, Framingham and SCORE models to Newcastle Heart Project data,” *J. Public Health (Bangkok).*, vol. 27, no. 1, pp. 93–100, 2005.

[6] R. G. Nelson *et al.*, “Development of Risk Prediction Equations for Incident Chronic Kidney Disease,” *JAMA - J. Am. Med. Assoc.*, vol. 322, no. 21, pp. 2104–2114, 2019.

[7] C. M. O’Seaghdha *et al.*, “A risk score for chronic kidney disease in the general population,” *Am. J. Med.*, vol. 125, no. 3, pp. 270–277, 2012.

[8] K. L. Chien, H. J. Lin, B. C. Lee, H. C. Hsu, Y. T. Lee, and M. F. Chen, “A prediction model for the risk of incident chronic kidney disease,” *Am. J. Med.*, vol. 123, no. 9, pp. 836-846.e2, 2010.
